# Distinct *Taphrina* strains from the phyllosphere of birch exhibiting a range of witches' broom disease symptoms

**DOI:** 10.1111/1462-2920.16037

**Published:** 2022-05-17

**Authors:** Margaretta Christita, Timo P. Sipilä, Agate Auzane, Kirk Overmyer

**Affiliations:** ^1^ Organismal and Evolutionary Biology Research Program, Faculty of Biological and Environmental Sciences, and Viikki Plant Science Centre University of Helsinki Helsinki Finland; ^2^ Environment and Forestry Research and Development Institute of Manado, Jalan Adipura, Mapanget Manado North Sulawesi Indonesia

## Abstract

The phyllosphere is an important microbial habitat and reservoir of organisms that modify plant health. *Taphrina betulina* is the causal agent of birch witches' broom disease. *Taphrina* species are dimorphic, infecting hosts in the filamentous form and residing in the host phyllosphere as non‐infectious yeast. As such, they are expected to be found as resident yeasts on their hosts, even on healthy tissues; however, there is little experimental data supporting this supposition. With the aim of exploring the local infection ecology of *T*. *betulina*, we isolated yeasts from the phyllosphere of birch leaves, using three sample classes; infected leaves inside symptom‐bearing branches, healthy leaves from symptom‐free branches on symptom‐bearing trees and leaves from symptom‐free branches on symptom‐free trees. Isolations yielded 224 yeast strains, representing 11 taxa, including *T*. *betulina*, which was the most common isolate and was found in all sample classes, including symptom‐free samples. Genotyping revealed genetic diversity among these *T*. *betulina* isolates, with seven distinct genotypes differentiated by the markers used. Twenty‐two representative *T*. *betulina* strains were selected for further study, revealing further phenotypic differences. These findings support that *T*. *betulina* is ubiquitous on birch and that individual trees host a diversity of *T*. *betulina* strains.

## Introduction

The phyllosphere has been recognized as an important habitat for microorganisms for over a century (Vorholt, 2012). Microbes residing in the phyllosphere have various lifestyles and interactions with their hosts ranging from mutualistic symbionts, commensal residents, to pathogens (Vorholt, 2012; Rastogi *et al*., 2013). Various yeast‐like fungi have been reported as resident in different plant compartments including the phyllosphere (Fonseca and Inácio, 2006; Yurkov *et al*., 2015; Begerow *et al*., 2017; Kemler *et al*., 2017; Limtong and Nasanit, 2017), some of which can modulate plant health both by directly interacting with the host and by reshaping the microbiome (Agler *et al*., 2016; Regalado *et al*., 2020; Brachi *et al*., 2021).

*Taphrina* are phytopathogenic yeasts causing disease often involving tumour symptoms mostly on woody plant species. These organisms have a dimorphic lifestyle, frequently residing in the host phyllosphere for long periods in their haploid budding yeast form and invading their hosts in their pathogenic dikaryotic filamentous form when environmental conditions are favourable (Mix, 1949; Fonseca and Rodrigues, 2011). *Taphrina* species, including *T*. *betulina* (Kern and Naef‐Roth, 1975), are able to produce the plant hormones auxin and cytokinin (Cissé *et al*., 2013; Streletskii *et al*., 2016; Wang *et al*., 2016; Streletskii *et al*., 2019). Auxin and cytokinin are widely believed to be involved in the many tumour and leaf deformation symptoms caused by various *Taphrina* species, although this has not been well tested experimentally. Auxin is also an important adaptation in phyllosphere resident microbes (Fonseca and Inácio, 2006; Kemler *et al*., 2017) thus may be involved in multiple aspects of lifestyle in *Taphrina* species. The genus *Taphrina* belongs to the order Taphrinales along with its sister genus *Protomyces*, and other genera, all of which are plant pathogens with similar lifestyles and pathogenesis strategies. As members of the Ascomycota subphylum Taphrinomycotina, these yeasts possess many ancestral characteristics and thus are of considerable evolutionary interest (Wang *et al*., 2019; Wang *et al*., 2021). The genomes of several species in Taphrinales are now available, opening these organisms to the possibility of molecular studies (Cissé *et al*., 2013; Tsai *et al*., 2014; Wang *et al*., 2019; Wang *et al*., 2020).

**Fig. 1:**
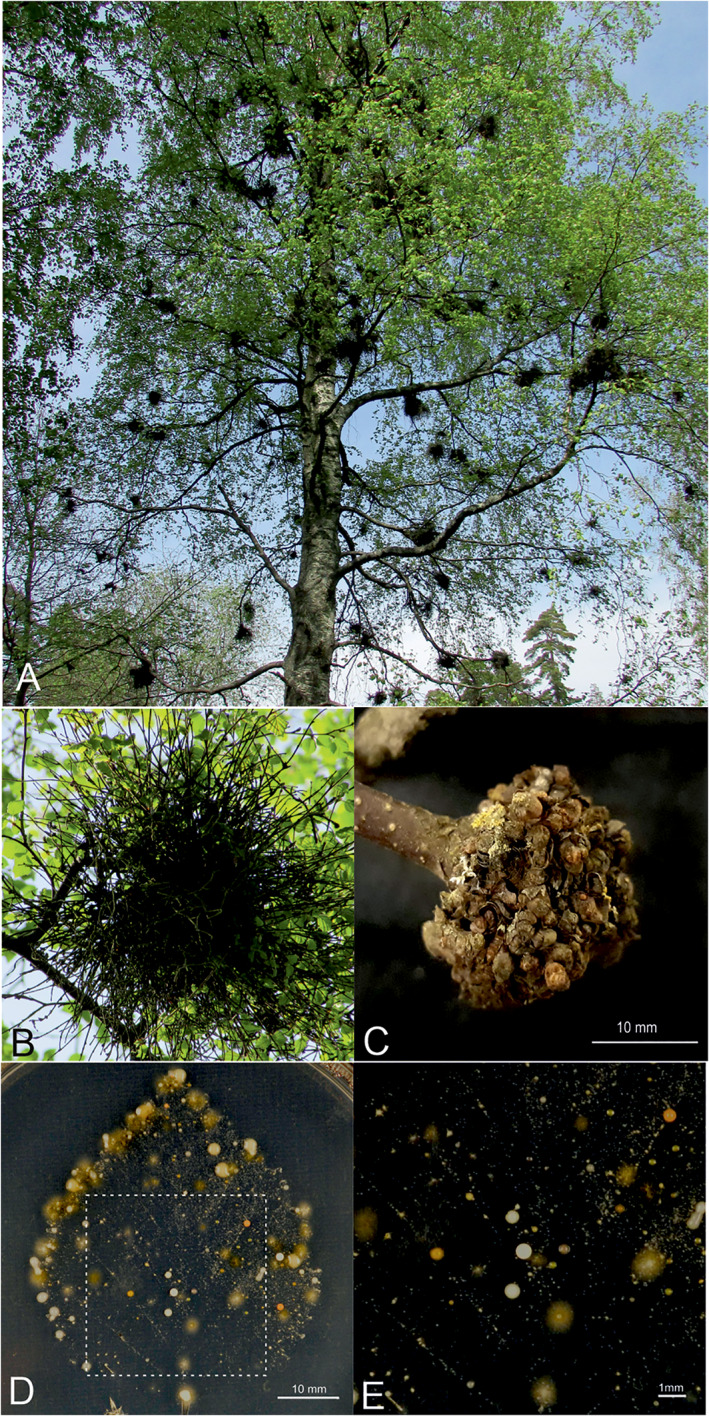
Witches' broom disease symptoms and leaf press culture. (A) Typical witches' broom symptoms on a heavily infected birch tree. (B) Detail of a typical broom with elongated shoots. (C) Detail of an atypical broom symptom, in which the central woody tumour is covered in buds that have not elongated into shoots. Size bar = 1 cm. (D). Birch leaf press culture demonstrating the presence of yeasts in the phyllosphere of *B*. *pendula*. Yeasts were cultivated for 14 days on 0.2× PDA. Size bar = 1 cm. (E). Close up details of the area marked with a box in (D) showing colonies with a typical yeast morphology, some of which are consistent with known colony morphology and cream colour of *T*. *betulina*. Size bar = 1 mm.

*Taphrina betulina* and several closely related *Taphrina* species (Table 1) are the causal agents of witches' broom disease on birch (*Betula* spp.), which induce distinctive broom symptoms (Fig. 1A and B) formed from proliferation of axillary buds and shoots around a central infected bud (Mix, 1949; Jump and Woodward, 1994; Fonseca and Rodrigues, 2011; Christita and Overmyer, 2021). Other symptoms include changes in leaves, such as enlargement (but not thickening), chlorosis, leaf curl, leaf spots, premature senescence and necrosis (Mix, 1949; Fonseca and Rodrigues, 2011). *Taphrina* species, including *T*. *betulina*, have most often been isolated from hosts with disease symptoms. Exceptionally, some *Taphrina* species, such as *T*. *inositophila* and *T*. *kurtzmanii*, have been isolated from disease‐free hosts (Inácio *et al*., 2004; Fonseca and Inácio, 2011).

**Table 1:** *Taphrina* species infecting *Betula* species^a^.

Species	Spore sizes (μm)	Species observed on
*Taphrina betulicola*	Spores 3.5 × 3	*Betula emani*
*T*. *betulina* ^b^	Ascospores 4.5–6.5 × 4–5.5 Blastospores 3.5–6 × 2–4.5	*B*. *aurata*, *B*. *carpatica*, *B*. *intermedia* ^c^, *B*. *nana*, *B*. *pendula* and *B*. *pubescens*
*T*. *nana* ^d^	Ascospores 3.5–6 × 3.5–5	*B*. *emani*, *B*. *intermedia*, *B*. *japonica*, *B*. *nana*, *B*. *pendula* and *B*. *pubescens*
*T*. *americana*	Spores 4–6 × 3.5–5 Blastospores 2.9–4 × 5.7–7.6	*B*. *fontinalis*, *B*. *lutea* and *B*. *papyrifera*
*T*. *boycei*	Ascospores 4–5 × 3.5–4	*B*. *fontinalis* and *B*. *occidentalis*
*T*. *carnea* ^e^	Ascospores 5–6 × 3–4 Blastospores 3–6 × 2–4	*B*. *fruticose*, *B glandulosa*, *B*. *humilis*, *B*. *intermedia*, *B*. *lutea*, *B*. *nana*, *B*. *papyrifera*, *B*. *pendula* and *B*. *pubescens*
*T*. *bacteriospermia*	Blastospores 3–6 × 1–2	*B*. *glandulosa*, *B*. *intermedia*, *B*. *nana* and *B*. *pubescens*
*T*. *betulae*	Ascospores 4–6 × 3.5–5	*B*. *intermedia*, *B*. *medwediewi*, *B*. *pendula* var. *purpurea*, *B*. *pubescens* and *B*. *turkestanica*
*T*. *flava*	Blastospores 5–6 × 5–5.5	*B*. *papyrifera and B*. *populifolia*

^a^ Compiled from Mix (1949) and Fonseca and Rodrigues (2011).

^b^ Includes the species *Exoascus betulinus*, *E*. *turgidus*, *T*. *turgida*, *T*. *willeana*, *T*. *lapponica*, *E*. *lapponicus*, *T*. *lagerheimii* and *T*. *splendens* shown by Mix (1949) to be synonymous with *T betulina*.

^c^ *B*. *intermedia* is a hybrid (*B*. *nana* × *B*. *pubescens*).

^d^ *T*. *nana* has an ITS sequence and PCR fingerprint identical to *T*. *betulina* (Rodrigues and Fonseca, 2003) and includes the synonymous species *T*. *alpina* (Mix, 1949).

^e^ *T*. *carnea* has an ITS sequence and PCR fingerprint identical to *T*. *betulina* (Rodrigues and Fonseca, 2003) and includes the synonymous species *T*. *janus and T*. *lata* (Mix, 1949).

The impact of witches' broom disease is generally underappreciated and often regarded a curiosity more than a damaging disease (Price and Macdonald, 2012). However, the few studies that have addressed this issue illustrate the need to re‐evaluate this view. A negative impact of witches' broom disease on *B*. *pubescens* has been demonstrated, showing decreased stem quality, vigour and growth (Spanos and Woodward, 1994). In *T*. *betulina*‐infected *B*. *maximowicziana* leaves, reduced photosynthesis and dark respiration, chlorosis and leaf loss were reported (Koike and Tanaka, 1986). The presence of witches' brooms in birch trees was shown to cause rapid death of the branches where they form and to negatively alter crown architecture (Kostina *et al*., 2015). Consequently, this disease may result in production losses under short rotation forestry programs (McKay, 2011).

Birch is an important hardwood forest species of considerable economic and ecological importance (Hynynen *et al*., 2009; Zohren *et al*., 2016). It grows in cooler temperate climates and is prone to potential negative effects of climate change, making a better understanding of its biology and ecology essential. The genomes of several birch species are now available (Wang *et al*., 2013; Salojarvi *et al*., 2017). The utility of birch as a model woody forest species has been demonstrated, including the use of molecular studies and induced rapid flowering for forward genetic studies (Alonso‐Serra *et al*., 2019; Alonso‐Serra *et al*., 2020). These developments make birch an attractive model for the study of microbial interactions with long‐lived forest species.

Several yeast genera have been reported as common members of phyllosphere communities on multiple plant species, such as; *Rhodotorula*, *Cryptococcus*, *Sporobolomyces* and *Dioszegia* (Fonseca and Inácio, 2006). Previous studies have addressed the fungal endophytes (Helander *et al*., 2007), yeast associated with birch forests (Yurkov *et al*., 2015) and fungal phyllosphere residents of birch (Helander and Rantio‐Lehtimaki, 1990; Helander *et al*., 1993; Nguyen *et al*., 2017). Nguyen *et al*. (2017) have identified yeasts in the Taphrinales in the birch phyllosphere, but these were not identified to the species level. Thus, the basic ecology of *T*. *betulina* on birch remains uncharacterized. Here we isolate and identify resident yeasts, including *T*. *betulina*, from the *B*. *pendula* phyllosphere, with the goal of investigating the genetic and possible functional diversity of *T*. *betulina* strains in host trees exhibiting varied levels of witches' broom symptoms.

## Results

### Sample collection and yeast isolation from birch

To assess if *Taphrina*‐like yeasts could be found from the phyllosphere of asymptomatic birch leaves, leaf press cultures were performed using birch leaves collected in the field from asymptomatic trees. Following cultivation using media and conditions that favoured yeasts, a high diversity of yeast colonies were apparent (Fig. 1D). The leaf press culture revealed a large diversity of yeasts in the birch phyllosphere, including yeasts coloured with white, beige and orange to pink pigments, some of which are consistent with *T*. *betulina* (Fig. 1D and E).

This prompted us to isolate birch‐associated yeasts using a total of nine leaf samples (A–I) collected from both symptomatic and asymptomatic trees at five independent sites in eastern Helsinki (Table 2). Samples were classified into three types based on their health characteristics; type I was symptomatic branches from symptomatic trees, type II was asymptomatic branches from symptomatic trees and type III was asymptomatic branches from asymptomatic trees. Witches' broom disease on birch was manifest in distinctive types of broom symptoms; typical brooms had multiple elongated shoots that had grown from many ectopic axillary buds, which formed around the primary infected bud, to form a central tumour (Fig. 1B). Occasionally, infected branches were found with a central tumour covered in buds that had not elongated into shoots (Fig. 1C). There was also a continuum of variation in the length of the shoots, in between these two extremes. To address the possibility that different *Taphrina* strains may be associated with these various structures, the phenotype of collected type I samples was further classified as elongated brooms (EB), short elongated brooms (SE), or tumour‐like (TL), while sample types II and III, which lacked broom symptoms, were classified as no broom (NO) (Tables S2 and 4). Broom morphology was specific to individual trees; i.e. each sampled individual had a single broom morphology type.

**Fig. 2:**
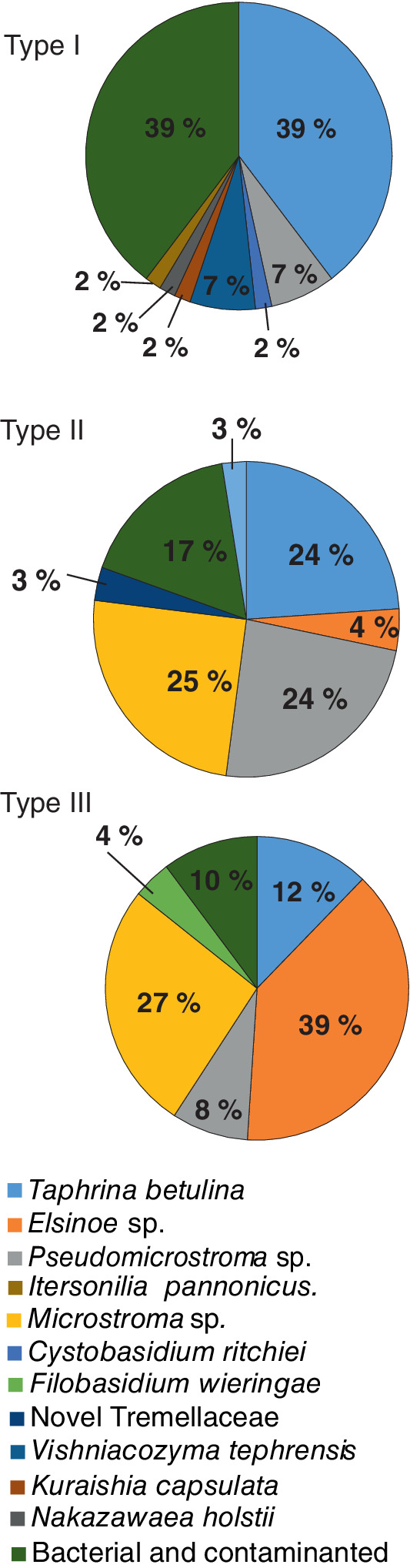
Identification of yeasts isolated from the phyllosphere of birch leaves. The identified yeasts were isolated from the birch phyllosphere from three sample types. Leaves were sampled from the following (type I, symptomatic branch from a symptomatic tree; II, asymptomatic branch from a symptomatic tree; III, asymptomatic branch from an asymptomatic tree), and are presented as percent of the total isolates in each of the three respective samples.

A total of 224 yeast strains were isolated from the birch phyllosphere (Table 2); with 58, 177 and 49 strains derived from type I, II and III samples respectively. Nuclear rRNA internal transcribed sequences (ITS) PCR products were analysed with the ITS Taq1 CAPS (ITC, i.e. digestion of the ITS PCR product with TaqI) marker, where identical banding patterns were binned and the ITS of representative strains were sequenced for molecular identification (Table S1). Of the 224 strains obtained (Table S2), 173 strains were identified in 11 taxa (Fig. 2; Table 3; Table S2); seven defined at the species level, namely *Taphrina betulina*, *Cystobasidium ritchiei*, *Filobasidium wieringae*, *Vishniacozyma tephrensis*, *Itersonilia pannonica*, *Kuraishia capsulata* and *Nakazawaea hosltii*; and four defined only to higher‐level classifications, specifically, one each in the genera *Pseudomicrostroma*, *Microstroma* and *Elsinoe*, and one in the family Tremellaceae (Fig. S2A; Tables 3 and S2). The remaining 51 strains were excluded from further analysis; 33 did not amplify an ITS PCR product and microscopic examination of a subset confirmed that they were prokaryotes, and 18 had very poor ITS PCR amplification or multiple ITS bands indicating they were contaminated (Table S1). The number of isolates was similar in all samples (Table 2), with two exceptions. Sample H′ was not a true independent sample, but a control that was identical to sample H, except that it was surface sterilized and sliced prior to the leaf wash step, which was done to control for endophytic yeasts. The low number of isolates in this sample suggests that the overwhelming majority of yeasts isolated in this study were epiphytic, i.e. phyllosphere residents. The number of leaf samples for each sample type varied (3, 5 and 2 for types I–III respectively; Table 2). To account for this sampling bias and facilitate comparisons between samples of differing levels of witches' broom disease symptoms, the normalized number of isolates (average number of isolates per sample type) is presented (in parentheses; Table 3).

**Table 2:** Sampling sites and leaf samples collected^a^.

Collection site^b^	GPS coordinates	Symptoms^c^		Sample	Strains isolated by sample type^d^			
		Tree	Branch		I	II	III	Total
Pihlajisto	60.230525 N, 24.996874 E	−	−	A	−	−	29	29
Viikki	60.226432 N, 25.012952 E	+	+	B	31	−	−	56
		+	−	C	−	25	−	
Vartioharju	60.218087 N, 25.118297 E	+	−	D	−	18	−	45
		+	+	E	27	−	−	
Vartiokylä	60.219020 N, 25.101769 E	−	−	F	−	−	20	42
		+	−	G	−	22	−	
Herttoniemi	60.201161 N, 25.043272 E	+	−	H	−	22	−	52
		+	−	H′^e^	−	2	−	
		+	−	I	−	28	−	
Total					58	117	49	224

^a^ All sampling was done in early August 2012 (samples A–C on August 3rd and samples D–I on August 7th). For list of isolated strains see Table S2.

^b^ All samples were collected from five different sites in eastern Helsinki, sites listed are districts within Helsinki.

^c^ Tree and branch health status for witches' broom symptoms; asymptomatic described as (−) and symptomatic described as (+).

^d^ Type I, symptomatic branches from symptomatic tree; type II, asymptomatic branches from symptomatic tree; type III, asymptomatic branches from asymptomatic tree.

^e^ Sample H′ is and identical to sample H except that it was first surface sterilized then cut into pieces prior to the leaf washing step. This served as a control for isolation of endophytic fungi.

**Table 3:** Yeasts isolated from the birch phyllosphere^a^.

Species	Type I^c^	Type II^d^	Type III^e^	Total
***T*. *betulina* genotypes** ^b^				
ITC‐C RGR‐0	1 (0.5)	11 (2.2)	–	
ITC‐C RGR‐1	–	–	1 (0.5)	
ITC‐C RGR‐2	–	1 (0.2)	–	
ITC‐D RGR‐0	8 (4)	1 (0.2)	1 (0.5)	
ITC‐D RGR‐1	–	1 (0.2)	–	
ITC‐D RGR‐2	14 (7)	11 (2.2)	4 (2)	
ITC‐D RGR‐3	–	3 (0.6)	–	
*T*. *betulina* total	23 (11.5)	28 (5.6)	6 (3)	57
**Other isolates**				
*Elsinoe* sp.	–	5 (1)	19 (9.5)	
*Pseudomicrostroma* sp.	4 (2)	28 (5.6)	4 (2)	
*Microstroma* sp.	–	29 (5.8)	13 (6.5)	
*Cystobasidium ritchiei*	1 (0.5)	–	–	
*Filobasidium wieringae*	–	–	2 (1)	
*Vishniacozyma tephrensis*	4 (2)	–	–	
*Kuraishia capsulata*	1 (0.5)	–	–	
*Nakazawaea holstii*	1 (0.5)	–	–	
*Itersonilia pannonica*	1 (0.5)	–	–	
Novel Tremellaceae	–	4 (0.8)	–	
Bacterial or contaminated	23 (11.5)	23 (4.6)	5 (2.5)	
Other isolates total	35 (17.5)	89 (17.8)	43 (21.5)	167
Total isolates				224

^a^ Table shows the number of independent isolates for each species or for each classification of *T*. *betulina* strains. Species were identified based on nuclear rRNA ITS gene sequences. Due to the different number of samples collected per sample type, the number of isolates per sample type normalized to eliminate bias from the different number of samples is in parentheses to facilitate comparisons. For further details on CAPS markers see Fig. S3. Diversity of *T. betulina* strains per sample type was as follows; type I, 3 (1.5); type II, 6 (1.5); type III 3 (1.5). For other species isolated, the diversity of species per sample type was as follows; type I, 8 (4); type II, 5 (1); type III 5 (2.5).

^b^ *Taphrina betulina* genotypes are defined by two CAPS markers. ITC (ITS Taq I CAPS) uses ITS PCR products digested with the restriction endonuclease Taq I and two ITC types (ITC‐C and ITC‐D) were found for *T*. *betulina*. RGR (*Rco1 Gyg7* RsaI) is a CAPS marker that amplifies the polymorphic intergenic region between the *T*. *betulina Gyg7* and *Rco1* genes and is digested with the restriction nuclease RsaI. It is used to differentiate between *T*. *betulina* strains and identifies four different banding patterns RGR‐0, no PCR product, and RGR‐1 to RGR‐3, which represent banding pattern variants I–III respectively (Table S3). Yeast species were isolated from three classes of birch leaf samples.

^c^ Type I, symptomatic branches from symptomatic tree.

^d^ Type II, asymptomatic branches from symptomatic tree.

^e^ Type III asymptomatic branches from an asymptomatic tree.

The dominant isolate, with a total of 57 strains (26% of all isolates) was *T*. *betulina* (Table 3). *Taphrina betulina* was present on all sample types, indicating this species can be isolated from disease‐free birch leaves. Additional analysis of *T*. *betulina* strains is further discussed below. Other yeast strains found in large numbers were the novel isolates of the genera *Elsinoe*, *Pseudomicrostroma* and *Microstroma*. Notably, although the level of diversity of yeast present was fairly constant, some differences in the yeast species isolated were observed between the samples differing in their level of witches' broom disease symptoms. Novel isolates in the genus *Elsinoe* were dominant in completely asymptomatic trees (type III samples). Isolates in the genus *Pseudomicrostroma* were found on all three sample types, while *Microstroma* was prevalent in asymptomatic samples, both asymptomatic branches from broom bearing trees and trees with no brooms (type II and III host samples). In spite of their presence in high numbers on healthy leaves, *Microstroma* sp. isolates were absent from leaves in witches' brooms. A general trend was seen where the total number of isolates for species other than *Taphrina* was lower in more diseased tissue, while the number of *T*. *betulina* isolates exhibited the opposite trend (Fig. 2; Table 3).

### *Taphrina betulina* identification and characterization

ITS sequences were sufficient to identify the isolated *T*. *betulina* strains to the species level; however, there were no lineage‐specific secondary phylogenetic markers able to resolve different strains of this species. Accordingly, ITS CAPS analysis of the *T*. *betulina* strains isolated here only identified two different banding patterns (ITC‐C and ITC‐D; Fig. S3; Tables S1 and S2). The genotype ITC‐D had an ITS sequence identical to *T*. *betulina*. Also, *T*. *betulina*, *T*. *nana* and *T*. *carnea* had identical ITS sequences (Fig. S3G; Rodrigues and Fonseca, 2003). These *Taphrina* were previously isolated as distinct species originating from different birch species, but later proposed to be conspecific based on molecular analysis (Rodrigues and Fonseca, 2003). We utilized these and other closely related *Taphrina* species to develop a marker that can differentiate between strains of *T*. *betulina* and related species. Using the assembly of *T*. *deformans* genome (Cissé *et al*., 2013) and our unpublished *T*. *betulina* genome data (PRJNA188318), two pairs of adjacent conserved housekeeping genes (*Rco1*‐*Gyp7* and *Sad1‐Rax1*) were identified to have conserved synteny between *T*. *betulina* and *T*. *deformans*. Two sets of outward‐facing primers were designed within conserved domains of the gene pairs, allowing amplification of the polymorphic intergenic regions (Fig. S3B). Both primer pairs gave PCR products of the expected size with *T*. *betulina* genomic DNA; however, amplification with *T*. *deformans* DNA only occurred with the primers spanning the *Rco1* and *Gyp7* genes, suggesting they were better suited for marker development (Fig. S3C and D). Further tests demonstrated that these primers were able to amplify genomic DNA from all the tested birch‐associated *Taphrina* species, except *T*. *americana* (Fig. S3E). This PCR product digested with RsaI was used as an RFLP marker, which was able to distinguish between the related alder pathogen *T*. *robinsoniana* and the three *T*. *betulina* strains with identical ITS sequences (Fig. S3D). This marker was named RGR1 (*Rco1 Gyp7* RsaI) and was used to characterize *T*. *betulina* isolates. The ITC marker identified two variants (ITC‐C and ITC‐D) within the isolated *T*. *betulina* strains (Tables 3, S1 and S2). These were further genotyped using the RGR1 marker, which identified four variants (RGR‐0, no PCR product; and RGR‐1 to RGR‐3, banding pattern variants I–III respectively; see Table S3; Fig. S3E and F). Taking both the ITC and RGR markers into account, seven distinct genotypes were identified within our *T*. *betulina* isolates (Tables 3 and S2).

A stable level of strain diversity across all host sample types was seen for *T*. *betulina* (Table 3). For the other isolated yeast species diversity was higher in the leaves sampled in brooms, compared to healthy leaves from both healthy or symptom bearing trees (Table 3). In all cases the strains/species present were different.

The *T*. *betulina* strain ITS‐D RGR‐2 was dominant in all host types and followed the general trend of increasing levels in more diseased tissues. There were no clear differences in the *T*. *betulina* strains present on samples with different broom morphologies; however, some strain genotypes only present in low numbers may be specific (Table 4). The distribution of strains was quite varied with some specific to only diseased or healthy samples and others present on all sample types.

**Table 4:** *Taphrina betulina* strain diversity found on samples with different broom morphology types.^a^

*T*. *betulina* strains	NB	EB	TL	SB	Total
ITC‐C RGR‐0		2		10	12
ITC‐C RGR‐1	1				1
ITC‐C RGR‐2		1			1
ITC‐D RGR‐0	1	9			10
ITC‐D RGR‐1		1			1
ITC‐D RGR‐2	4	23	1	1	29
ITC‐D RGR‐3		1		2	3
Total	6	37	1	13	57

^a^ Table shows the number of independent isolates for each classification of *T*. *betulina* strains. *Taphrina betulina* genotypes are defined by two CAPS markers. ITC (ITS Taq I CAPS) uses nuclear rRNA ITS PCR products digested with the restriction endonuclease Taq I and two ITC types (ITC‐C and ITC‐D) were found for *T*. *betulina*. RGR (*Rco1 Gyg7* RsaI) is a CAPS marker that amplifies the polymorphic intergenic region between the *T*. *betulina Gyg7* and *Rco1* genes and is digested with the restriction nuclease RsaI. It is used to differentiate between *T*. *betulina* strains and identifies four different banding patterns RGR‐0, no PCR product, and RGR‐1 to RGR‐3, which represent banding pattern variants I–III respectively (Table S3). Abbreviations used: NB, no broom; EB, elongated broom; TL, tumour‐like; SB, short broom.

**Fig. 3:**
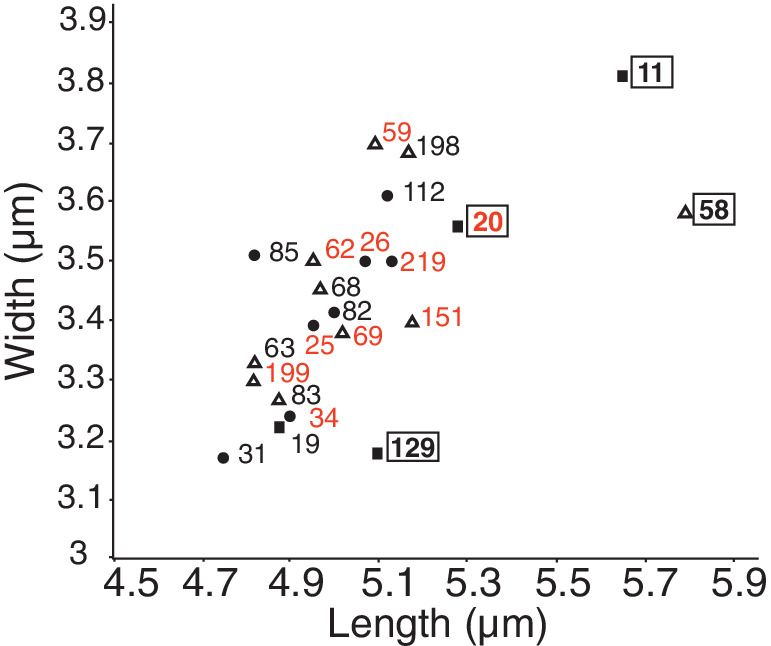
Scatter plot of *T*. *betulina* strain cell size. Cell sizes for the 22 selected *Taphrina betulina* strains. Each strain number is given next to the data points. Sample type from which the strain originated is indicated by the data point shape as follows: circles, type I samples (symptomatic branch from symptomatic trees); triangles, type II samples (asymptomatic branch from symptomatic trees); squares, type II samples (asymptomatic branch from asymptomatic trees). Numbers enclosed in a box indicate strains whose cell sizes were significantly different from those of other strains (see Table S4) and numbers depicted in red represent strains of the genotype ITC‐D RGR‐2.

We selected 22 of 57 strains of *T*. *betulina*, which were representative of the diversity in the collection in terms of their genotype and sample origin (Table 5). Half of the 22 selected strains originated from type II, while type I was 32% and type III was 18% (Fig. S2) and all genotypes were represented (Table 5). Additionally, given the limitations of the markers used in genotyping, it is expected that additional unseen diversity may be found in these isolates. Accordingly, some redundant samples were selected for further analysis in order to test for phenotypic diversity. This strain collection was used for further identification and characterization. First, the morphology of the selected strains was characterized. Colonies of 22 selected strains streaked on 0.2× PDA exhibited variation in colour from pale pink to peach (Fig. S4, inset), while yeast cells did not exhibit any notable morphological differences (Fig. S4). The average yeast cell sizes of all strains taken together were within the range of 3.17–3.81 × 4.75–5.79 μm (Table S4). For comparison, the yeast cell sizes for birch‐associated *Taphrina* species are listed in Table 1. Size variation between individual strains within the collection was observed in plots of the average length and width (Fig. 3; Table S4). ANOVA followed by Tukey's post hoc test showed the cell sizes of strains 11, 20, 58 and 129 were significantly different to other strains (Table S4; Fig. 3). There was one common feature to all these strains – they originated from healthy leaves (Table 5); strains 11, 20 and 129 were from completely healthy trees (type III samples), and strain 58 from leaves of healthy branches on a broom‐bearing tree (type II sample). All *T*. *betulina* strains genotyped as ITC‐D RGR‐2 grouped together in the size plot indicating they have similar cell sizes.

**Table 5:** Representative *Taphrina betulina* strains selected for in‐depth analysis.

No.	Strain	Origin^a^	Growth^b^		ITC type^c^	RSR type^d^	Broom type^e^	Culture collection accession numbers^f^
			30°C	21°C				
Type I: Symptomatic branches from symptomatic trees								
I	25	B	−	+++	ITC‐D	RGR‐2	EB	FBCC 2711 = DSM113951
2	26	B	−	+++	ITC‐D	RGR‐2	EB	FBCC 2713 = DSM113952
3	31	B	−	+++	ITC‐D	RGR‐0	EB	FBCC 2729 = DSM113953
4	34	B	−	+	ITC‐D	RGR‐2	EB	FBCC 2715 = DSM113954
5	85	B	−	+++	ITC‐C	RGR‐0	EB	FBCC 2725 = DSM113950
6	112	E	+	+++	ITC‐D	RGR‐0	EB	FBCC 2732 = DSM113964
7	219	E	−	+++	ITC‐D	RGR‐2	EB	FBCC 2714 = DSM113965
Type II: Asymptomatic branches from symptomatic trees								
8	58	C	−	+++	ITC‐D	RGR‐3	EB	FBCC 2716 = DSM113955
9	59	C	−	+++	ITC‐D	RGR‐2	EB	FBCC 2720 = DSM113956
10	62	C	−	+++	ITC‐D	RGR‐2	EB	FBCC 2727 = DSM113957
11	63	C	−	+	ITC‐C	RGR‐0	EB	FBCC 2731 = DSM113949
12	68	C	+	+++	ITC‐C	RGR‐2	EB	FBCC 2723 = DSM113948
13	69	C	+	+++	ITC‐D	RGR‐2	EB	FBCC 2724 = DSM113958
14	82	C	−	+++	ITC‐D	RGR‐1	EB	FBCC 2717 = DSM113959
15	83	C	−	+++	ITC‐D	RGR‐0	EB	FBCC 2721 = DSM113960
16	151	G	−	+++	ITC‐D	RGR‐2	TL	FBCC 2730 = DSM113963
17	198	I	−	+++/+	ITC‐D	RGR‐3	SE	FBCC 2726 = DSM113961
18	199	I	−	+++/+	ITC‐D	RGR‐2	SE	FBCC 2728 = DSM113962
Type III: Asymptomatic branches from asymptomatic trees								
19	11	A	−	+	ITC‐C	RGR‐1	NO	FBCC 2718 = DSM113944
20	19	A	−	+++	ITC‐D	RGR‐0	NO	FBCC 2719 = DSM113946
21	20	A	−	+++	ITC‐D	RGR‐2	NO	FBCC 2722 = DSM113945
22	129	F	−	+++	ITC‐D	RGR‐2	NO	FBCC 2712 = DSM113947

^a^ Samples are defined in Table 2.

^b^ Strain growth was measured by drop inoculations of serial dilutions on 0.2× PDA and incubation at the indicated temperature for 4 days then were visually assessed and characterized as noted: +++, strong growth; ++, intermediate growth; + weak growth.

^c^ Nuclear rRNA gene ITS PCR products were used as CAPS marker, called ITS Taq I CAPS (ITC), by digestion with the restriction endonuclease Taq I. ITC types detected in *T*. *betulina* strains were ITC‐C and ITC‐D.

^d^ RGR (*Rco1 Gyg7* RsaI) is a CAPS marker that amplifies the polymorphic intergenic region between the *Taphrina betulina Gyg7* and *Rco1* genes and is digested with the restriction nuclease RsaI. It is used to differentiate between *T*. *betulina* strains and identifies four different banding patterns RGR‐0, no PCR product, and RGR‐1 to RGR‐3, which represent banding pattern variants I–III respectively. See Fig. S2 for information on the CAPS markers.

^e^ Broom symptoms caused by *T*. *betulina* were varied in their morphology, primarily in the length of broom twigs and were classified as follows: EB, elongated brooms for brooms with twigs of usual length; SB, short brooms, for brooms with shorter twigs; TL, tumour‐like, for brooms in which buds formed around the central tumour, but did not elongate. For example brooms, see Fig. 1B and C.

^f^ These strains are deposited in the following culture collections where they are maintained in a metabolically inactive state and available under the given accession numbers. FBCC, The University of Helsinki Microbial Domain Biological Resource Centre (HAMBI) fungal collection; DSM, the German Collection of Microorganisms and Cell Cultures GmbH (DSMZ).

**Fig. 4:**
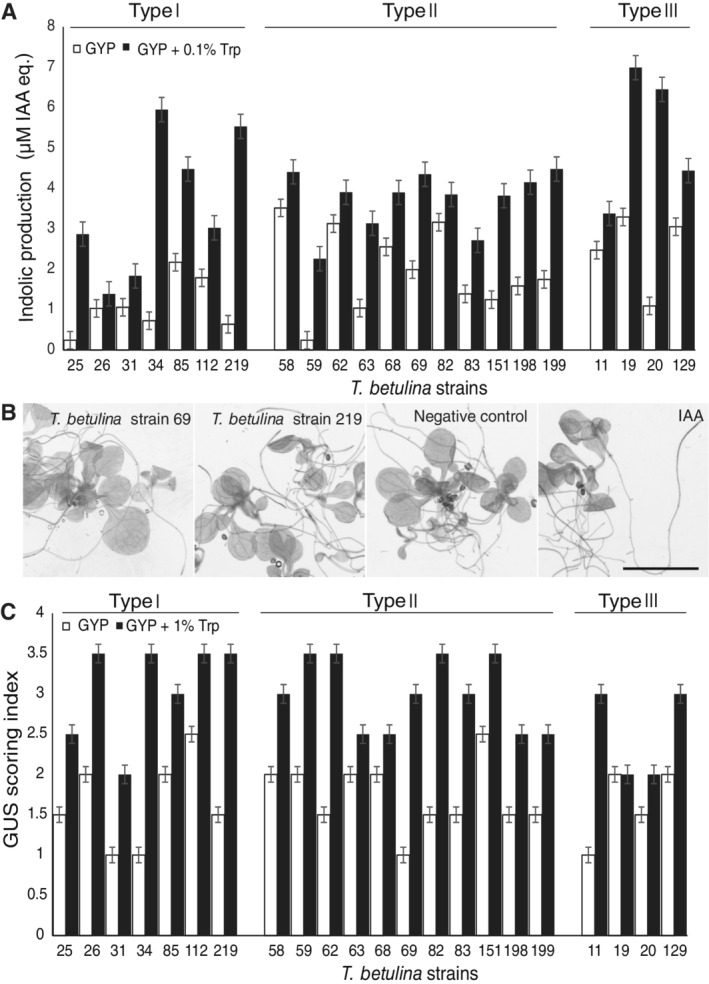
Activation of plant auxin response by *Taphrina betulina* culture filtrates. (A) Indolic compound production was used as a proxy for auxin production and was assayed spectrophotometrically using the Salkowski assay. *Taphrina betulina* strains (22 total) were cultivated in YPD (yeast extract, peptone, dextrose) with and without 0.1% tryptophan. Three independent biological repeats each with three technical replicates were done for each individual strain. Production levels were calibrated according to a standard curve with the auxin, IAA and are expressed as IAA equivalents. (B) The activation of *Arabidopsis* auxin transcriptional response by *T*. *betulina* culture filtrates was monitored as expression of the auxin‐responsive DR5 promoter fused to the β‐glucuronidase (GUS) reporter gene. Two‐week‐old *in vitro* grown DR5::GUS *Arabidopsis* seedlings in 0.5 MS media were treated with filtered supernatants of 5‐day‐old yeast culture in YPD and YPD + 0.1% tryptophan for 24 h. Representative results are shown here: light background root tip staining was seen in strain 69 cultured in YPD, Fresh sterile YPD + 0.1% tryptophan was used as a negative control. A typical strong response was seen in strain 219 cultured in YPD + 0.1% tryptophan and treatment with 5 μM IAA was used as a positive control. For full results from all strains see Fig. S5. Scale bar = 1 cm and is valid for all micrographs. (C) DR5::GUS staining results in Fig. S5 were visually scored using an arbitrary GUS scoring index on a scale from 0 to 4. The representative responses shown in (B) were used for reference; where 0 is defined by the negative control and 4 by strain 219. Results from two independent biological repeats were scored and averaged.

The Salkowski assay was utilized to quantify production of indolic compounds, used here as a proxy for auxin production. The selected *T*. *betulina* strains were cultivated in two different media; YPD and YPD + 0.1% tryptophan, which is the biosynthetic precursor of indole acetic acid (IAA), the most important of the auxins in plants. All strains were able to produce indolic compounds in both media (Fig. 4). Indolic compound production was higher for all strains when cultivated in YPD supplemented with tryptophan, compared to YPD medium alone (Fig. 4). There were marked differences in indolic compound production between strains (Fig. 4A); however, this did not correlate with the sample type of origin, with both high and low producers found in individuals from all three sample types.

In order to gain further evidence for possible differences in auxin production by *T*. *betulina* strains, a plant‐based auxin‐response reporter system was used. *Arabidopsis* seedlings transgenically bearing the auxin‐responsive DR5 promoter fused to the β‐glucuronidase (GUS) reporter (DR5::GUS) were treated with yeast culture supernatants, then GUS histochemical staining was used to visualize tissues exhibiting activation of the plant auxin transcriptional response. Representative strains exhibiting low and high levels of GUS staining (strains 69 and 219 respectively) are shown (Fig. 4B) along with the negative control (fresh sterile media) and positive control (5 μM IAA). Photos of all treatments (Fig. S5) were visually scored on an arbitrary scale from 0 (no staining) to 4 (high staining) and are summarized in Fig. 4C. Treatment with culture supernatants of *T*. *betulina* strains resulted in activation of plant auxin response, to varied levels (Fig. 4C; Fig. S5). Strains isolated from type III samples exhibited lower staining levels suggesting a lower capacity to produce auxin. However, this was only apparent with culture filtrates from cells cultivated with exogenous tryptophan; there were no remarkable differences among samples cultivated in YPD, which represents more natural conditions (Figs 4C and S5).

## Discussion

*Taphrina* are enigmatic and little‐studied phytopathogenic yeast‐like fungi. Many of the basic details of *Taphrina* biology remain unknown or are little studied with modern methods. There is a large body of literature from previous studies of *Taphrina* species (Mix, 1949; Fonseca and Rodrigues, 2011). However, most of this work is quite outdated and even the known aspects of *Taphrina* biology warrant revalidation with modern methods. Here we have addressed the local distribution of *T*. *betulina* strains with emphasis on the distribution between and within birch branches presenting differing levels of witches' broom disease symptoms. Such work had not previously been undertaken and has implications for several aspects of *T*. *betulina* biology.

### Isolation of *T*. *betulina* from uninfected tissues

*Taphrina* species, including *T*. *betulina*, have been most commonly isolated from symptomatic host leaves (Mix, 1949) and have only infrequently been isolated from healthy tissues (Inácio *et al*., 2004; Fonseca and Inácio, 2011). *Taphrina betulina* has typically been isolated by the spore drop method from infected leaves with ascogenous cells breaking through the leaf surface (Mix, 1949; Tavares *et al*., 2004). Importantly, this most often associated a single isolate with a diseased host individual and has even led to the naming of new species based on the host of origin (Table 1). In this study, the use of modern high‐throughput isolation utilizing rapid PCR‐based molecular identification methods has facilitated the isolation of *T*. *betulina* from both symptom bearing material and healthy leaves. The molecular markers utilized in this work allowed differentiation of seven distinct genotypes. This indicates that, on a local scale, and even on individual birch trees, multiple distinct strains are present. Some of these strains may even represent closely related strains that were previously described as distinct birch‐associated *Taphrina* species, as is further discussed below. There was little evidence of apparent specificity to the strains present or their diversity within the three sample types examined. Furthermore, there were only few examples of specific strains associated with brooms exhibiting different morphologies. However, there is enough suggestion to warrant hypothesis building in order to guide future work. Our findings suggest the possibility of *T*. *betulina* strains with different lifestyles, such as non‐pathogenic phyllosphere residents, as are further discussed below, and indicate the need for further investigation.

The exact function of witches' broom structures caused by *T*. *betulina* infection remains unknown. This perennial infection symptom is most likely a favourable living space within the remodelled host that promotes survival of the pathogen. A feature of *Taphrina* that differentiates them from other Ascomycota is their naked asci that lack ascomata (Mix, 1949; Fonseca and Rodrigues, 2011). Thus, different broom structures may function as fruiting body‐like structures co‐opted from the host and could facilitate for instance wind dispersal of spores in different environments. This suggests the pathogen is driving broom morphology and predicts factors such as effector proteins or plant hormones produced by the microbe as candidates involved. Our findings do not support the involvement of auxin production; however, *Taphrina* also produce other plant hormones such as cytokinins and abscisic acid (Cissé *et al*., 2013; Streletskii *et al*., 2019), which may be involved. If no further evidence of structure‐specific strains is found, the alternative hypothesis is that the length of shoot elongation within brooms caused by *T*. *betulina* is then dependent on the host genotype, or other unknown factors, rather than the *T*. *betulina* strain present.

*Taphrina betulina*, like all *Taphrina* species, is dimorphic, infecting its host in the dikaryotic hyphal form in the spring and early summer, but existing as a phyllosphere resident in its yeast form for most of the year. Additionally, *Taphrina* species are sensitive to environmental conditions and only infect when favourable cold and wet conditions prevail during bud break in the spring (Giosuè *et al*., 2000; Rossi *et al*., 2006; Rossi *et al*., 2007). Thus, infections do not occur every year and *Taphrina* species are thought to be able to survive in the phyllosphere in their yeast form indefinitely (Mix, 1949; Fonseca and Rodrigues, 2011). Interestingly, these characteristics are consistent with those of an opportunistic pathogen. As such they are expected to be found even on healthy tissues of their hosts; although this has been confirmed by little previous experimental evidence. Strains of *T*. *betulina* with the genotype ITS‐D RGR‐2 were the most commonly found and exhibited a pattern where strains of this genotype were more frequently isolated from more diseased samples, making it a candidate for the being the primary cause of witches' broom disease in the trees studied here. Its presence on symptom‐free leaf samples supports the idea that virulent *T*. *betulina* strains can also be found as phyllosphere residents on apparently healthy trees. There are only a few studies that have previously made such observations, using molecular methods *T*. *deformans* has been detected from healthy peach trees (Tavares *et al*., 2004; Mikhailova *et al*., 2020). However, as is further discussed below, some strains specific to healthy trees have been found, which may have a non‐pathogenic phyllosphere resident lifestyle and whose host relationship remains uncharacterized. Indeed, the relationship of all phyllosphere resident *T*. *betulina* in its yeast form, including the disease‐causing strains, remains unknown. In general, the idea of possible dimorphic plant opportunistic pathogens remains underexplored. Future research might address the possibility that phyllosphere resident *T*. *betulina*, and other *Taphrina* species, may have beneficial effects for the host. This would be consistent with a previous study that suggests there is a high‐level diversity in birch susceptibility towards witches' broom disease, conferred by possible genetically encoded resistance loci segregating in wild populations (Christita and Overmyer, 2021) and would also account for the mixture of dominant and rare strains in healthy (types II and III) samples.

The presence of multiple strains in the same samples also suggests a diversity of *T*. *betulina* strains may be involved in the disease process. This is consistent with the known ability of *Taphrina* species to enter the infectious dikaryotic growth form by two distinct mechanisms; either with a single cell that undergoes nuclear duplication without cell division or by conjugation of two yeast cells. The former seems to be the dominant mechanism. The regulation of these varied sexual behaviours in *Taphrina* species is not well understood. Characterization of the *T deformans* genome indicates a MAT locus configured for primary homothallism (Almeida *et al*., 2015). Conjugation has only been rarely observed in a few species, *T*. *betulina* not among them (Mix, 1949). Further studies are required to explore the possibility of conjugation by *T*. *betulina* strains suggested by these findings.

Alternatively, some of the various strains present may have different specialist lifestyles. Some *Taphrina* species have been isolated from symptomless plants that were not previously known to be *Taphrina* hosts (Inácio *et al*., 2004; Fonseca and Inácio, 2011). Some species have no known host and have been isolated as endoliths, such as *T*. *antarctica* (Selbmann *et al*., 2014). These species have been found on multiple plant species, are thought to be non‐pathogens specialized in phyllosphere residency, and have broader than usual carbon utilization profiles (Inácio *et al*., 2004; Fonseca and Inácio, 2011). Strains of *T*. *betulina* with four genotypes (ITC‐C RGR‐1, ITC‐C RGR‐2, ITC‐D RGR‐1 and ITC‐D RGR‐3) were found only on healthy samples. Although, strains of these genotypes were also rare and further studies with deeper sampling may be required to understand their true distribution. Nonetheless, these strains raise the question of possible phyllosphere specialist *Taphrina* on birch. Examination of the carbon utilization capabilities of these strains would also help address this question.

### Isolated *T*. *betulina* strains compared to other *Taphrina* species isolated from birch

The practice of naming parasitic fungi after the host from which they have been isolated has been common for a large number of diverse fungi. Often single isolates are associated with disease phenotypes, without exploring the other strains that are present. In such cases, subsequent analyses frequently result in splits and mergers in species names, as is the case for *T*. *betulina*. There are currently many birch‐associated *Taphrina* species (Table 1). As is typical for all *Taphrina* species, *T*. *betulina* and the other birch‐associated *Taphrina* have a host range that is fairly wide within the genus *Betula* (Mix, 1949; Fonseca and Rodrigues, 2011). Some geographically separate species do exist; for instance, *T*. *americana* that is pathogenic on North American *Betula* species and *T*. *betulina* that is pathogenic on Eurasian *Betula* species. Very few of these birch‐associated *Taphrina* have been analysed with modern molecular methods and some have been lost to science with no viable cultures currently available.

A long‐running trend in research on birch‐associated *Taphrina* species has seen a reduction in their numbers through merging of conspecific lines (Mix, 1949; Christita *et al*., 2021). This calls for re‐evaluation of the many characteristics previously used to separate species, such as cell size, morphology, symptoms induced in the host, geographical location of isolation, host species, and so on. In their place, reliance on molecular studies must take precedence, with which further merging of conspecific strains can be expected. This is well illustrated by the cases of *T*. *carnea* and *T*. *nana*, which were originally separated from *T*. *betulina* based on combinations of morphology, host tissues infected and symptoms produced (Mix, 1949), but later proposed to be conspecific with *T*. *betulina* based on identical ITS sequences and PCR fingerprinting patterns (Rodrigues and Fonseca, 2003). However, confirmation of these results with additional strains of *T*. *nana* and *T*. *carnea* is still required before these species can be formally merged (Rodrigues and Fonseca, 2003; Fonseca and Rodrigues, 2011). Generally, the cell sizes for all of the 22 *T*. *betulina* isolates examined (Fig. 3; Table S4) were in agreement with the previously published results for *T*. *betulina*; 2.4–4 × 2–4 μm (Mix, 1949), 4.5–6 × 4–5 μm (Bacigalova, 1997), 4.5–5 × 6.7–8.2 μm (Fonseca and Rodrigues, 2011); however, some strains with distinct cell sizes were found (Table 1). In the order Taphrinales, which includes the genera *Taphrina* and *Protomyces*, ITS sequences are known to give phylogenetic resolution only to the genus level, or to the species level only for some taxa (Rodrigues and Fonseca, 2003; Wang *et al*., 2019; Wang *et al*., 2021). In the current work, we developed a new molecular marker named RGR that distinguishes between *T*. *betulina* strains. Remarkably, the use of this marker and the ITS sequence‐based CAPS marker ITC led to the detection of strains of ITC‐D RGR‐2 (Table 3), a genotype that was the most commonly isolated and has a genotype with these two markers that is identical to *T*. *nana* (Fig. S3). This finding suggests that the most commonly isolated *T*. *betulina* strain found in all sample types may be related to *T*. *nana*. The species *T*. *nana* has been previously observed on *B*. *pendula* (Table 1) (Mix, 1949; Fonseca and Rodrigues, 2011). To further support this observation, we compared the cell sizes of strains with the genotype ITC‐D RGR‐2 (Fig. 3; Table S4) to the published size ranges for *T*. *nana* (Table 1), which were roughly in agreement. Taken together these findings further support the synonymy of *T*. *nana* and *T*. *betulina*. However, examination of additional strains or further genetic comparisons between *T*. *betulina*, *T*. *nana*, and the ITC‐D RGR‐2 strains in this work will be required for confirmation.

There is clear evidence for birch‐associated *Taphrina* with distinct ITS sequences; *T*. *americana* has an ITS sequence that was different at 20 nucleotide positions (96.6% identical) compared to *T*. *betulina*, which supports that *T*. *americana* is a distinct species (Rodrigues and Fonseca, 2003; Vu *et al*., 2016) Strains of the genotype ITC‐C were also different from *T*. *betulina*, which had an ITS sequence identical to ITC‐D. Comparisons of ITS sequences indicate that ITC‐C is distinct from *T*. *americana* and most similar to *T*. *betulina*, differing only by six nucleotide positions (99.0% similar; Fig. S3G). This high level of similarity (>98.4%; Vu *et al*., 2016) supports that strains of the genotype ITC‐C are variants belonging to *T*. *betulina*.

Production of the indolic plant hormone auxin is a common feature of *T*. *betulina* (Kern and Naef‐Roth, 1975) and is known to have roles in phyllosphere residency, pathogenesis and possibly in tumour symptom formation (Fonseca and Inácio, 2006; Fu and Wang, 2011; Spaepen and Vanderleyden, 2011; Kemler *et al*., 2017). The birch‐associated *Taphrina* strains examined here exhibited large variation in the capacity to produce indole compounds and induce a plant auxin transcriptional response. However, these characters did not correlate with the sample of origin. Furthermore, there was no correlation between *Taphrina* strain genotype and auxin biology, with highly varied indolic compound producers or IAA responses found within the genotypes (Fig. 4; Table 5).

Finally, many *T*. *betulina* strains available in culture collections have been under long‐term *in vitro* cultivation or preservation. This study fulfilled the additional aim of collecting local strains of *T*. *betulina* for further study. The use of the 22 selected strains in this study for genome sequencing and other further studies will contribute to our understanding of the biology of *T*. *betulina*. The relationships between *Taphrina* strains isolated in this work and the known birch‐associated *Taphrina* species remain unclear. However, this work has produced important resources and begun the process of reconciling the older *Taphrina* literature with results using modern molecular methods. Importantly, in contrast to older work that associated a single *Taphrina* strain with an individual diseased tree, this work demonstrates a diverse population of different *Taphrina* strains on birch individuals.

### Other birch phyllosphere yeasts

Several other yeast species were also found in the birch phyllosphere, including isolates belonging to taxa commonly found in the phyllosphere of other plant species (Wang *et al*., 2016; Kemler *et al*., 2017). Several isolates in the genera *Pseudomicrostroma* and *Microstroma* were obtained; with *Pseudomicrostroma* found in all sample types and *Microstroma* associated only with healthy tissues. Species in these genera are known plant pathogens causing leaf spot diseases (Kijpornyongpan and Aime, 2017). Isolates in the genus *Elsinoe* were found in two independent sample types, using methods that favour the growth of yeasts, where only yeast‐like colonies were selected. Remarkably, the genus *Elsinoe* belongs to class Dothideomycetes in subphylum Pezizomycotina, which comprises filamentous Ascomycetes. Further examination of these isolates in the genus *Elsinoe* will be needed to determine if they are truly yeasts or yeast‐like fungi. Sequences belonging to Dothideomycetes have been previously found from birch leaves using high‐throughput sequencing of ITS2 libraries (Nguyen *et al*., 2017). Many species within the genus *Elsinoe* are known plant pathogens causing important anthracnose and scab diseases (Jayawardena *et al*., 2014); however, in the current study isolates in this phytopathogenic genus were only found on healthy birch samples, similar to the *Microstroma* isolates mentioned above.

*Filobasidium wieringae* and *Vishniacozyma tephrensis* were found in sample types I and III. Other isolates that were present in small numbers belonged to the genera *Cystobasidium*, *Vishniacozyma*, *Kuraishia*, *Nakazawae* and *Itersonilia*, which were only found in type I samples, and novel members of family Tremellaceae, which were only found in type II samples. Generally, the number of isolates of species other than *Taphrina* was higher in healthy tissues, while conversely, *T*. *betulina* isolates were more prevalent in diseased samples. For example, isolates from the genera *Microstroma* and *Elsinoe* were abundant in healthy samples but absent from leaves in witches' brooms. These findings suggest that *T*. *betulina* may have altered the phyllosphere microbiome within diseased tissues. Alternatively, difference in *B*. *pendula* genotypes in susceptible/resistant individuals may influence phyllosphere microbiome composition. Taken together these results are consistent with the concept of dysbiosis, an imbalance or alteration of microbial communities in diseased tissues (Chen *et al*., 2020). However, further studies will be required to explain the observed differences in resident yeasts in the phyllosphere of diseased and healthy birch.

This work is the starting point for molecular genetic studies of birch interactions with the yeast‐like pathogen *T*. *betulina*. Yeast‐like fungi have a wide range of effects on plant health. A deeper understanding of yeast interactions with forest species opens the possibility of developing improved and biologically based silviculture and forest conservation measures.

## Experimental procedures

### Sampling, isolation and cultivation of yeasts

Leaves of birch (*Betula pendula* Roth) were sampled from several locations around eastern Helsinki, Finland (Table 2), placed into sterile 50 ml centrifuge tubes (Sarstedt; www.sarstedt.com) and stored at +4°C prior to further processing. Leaf press cultures were prepared by placing birch leaves on the surface of a Petri dish containing 0.2× potato dextrose broth (PDB; BD Difco; www.fishersci.com) with 15 g L^−1^ agar (PDA), covered with sterile filter paper, and pressed with gloved fingertips to transfer phyllosphere microbes to the medium. Leaf press cultures were cultivated 1–2 days at 21°C and then stored at 4°C for 2 weeks to promote colony colour formation. For yeast isolation, three samples were collected for type I samples, which were symptomatic leaves (harvested from inside brooms) from symptomatic trees, five samples for type II samples, which were leaves from asymptomatic branches from symptomatic trees, and two samples were collected for type III samples, which were leaves from asymptomatic branches from asymptomatic trees. Leaf samples were gently rinsed three times in sterile ultrapure water. Leaves were placed in sterile 50 ml centrifuge tubes with 5 ml of sterile ultrapure water with 0.025% Silwet‐L77, then spores were released by vigorous agitation with a vortex mixer. A dilution series (10^−1^–10^−3^) of the leaf wash containing suspended microbial cells was plated on Petri dishes with 0.2× PDA and cultivated for 1 week at 21°C under a 12 h light cycle in a controlled environment chamber (Model MLR‐350, Sanyo, uk.vwr.com). Colonies with yeast‐like morphology were picked after 3, 5 and 7 days, and isolated by two rounds of streaking onto fresh 0.5× PDA supplemented with 100 μg ml^−1^ ampicillin. An equal number of Petri dishes made for each sample and the number of colonies picked represented the number and diversity of colonies present on the Petri dish. Wash solutions and isolated strains were placed in 50% sterile glycerol at −80°C for long‐term storage. For short‐term storage cells from each strain were inoculated into 200 μl sterile water in 96 well plates to facilitate high‐throughput inoculation, cultivation and DNA isolation.

### DNA extraction and yeast identification

Following cultivation in 2 ml 0.2× PDB for 5 days, cells were harvested by centrifugation (5 min × 12 000*g*) for DNA isolation as previously described (Looke *et al*., 2011). Briefly, cells were re‐suspended in one volume lysis buffer (100 mM Tris–HCl pH 8, 50 mM EDTA, 500 mM NaCl), then vortexed for 3 min with 0.3 g glass beads and 200 μl phenol/chloroform/isoamyl alcohol. After adding 200 μl TE, samples were briefly vortexed, then centrifuged for 5 min. The aqueous layer was transferred to a clean 2 ml tube, ethanol precipitated, pelleted, re‐suspended in 0.4 ml TE buffer, treated with DNase‐free RNase A (5 min at 37°C), ethanol precipitated and dissolved in 100 μl TE. Yeast strains were identified based on sequences of their internal transcribed spacer (ITS) region of the nuclear rRNA locus. ITS PCR products were amplified from yeast genomic DNA with the ITS3 (5′‐CTTGGTCATTTAGAGGAAGTAA‐3′) and ITS4 (5′‐TCCTCCGCTTATTGATATGC‐3′) primers as previously described (Wang *et al*., 2016). The cultivation media used in isolations also supports the growth of some prokaryotic species. Strains that did not give an ITS PCR product were suspected to be bacterial, some of which were screened microscopically initially and confirmed to be bacteria. Subsequently, all strains not producing an ITS PCR product were eliminated from further analysis. Isolates that gave multiple ITS PCR bands were considered contaminated and eliminated from further analysis. Morphologically similar yeasts with identical ITS PCR product lengths underwent cleaved amplified polymorphic sequence (CAPS) analysis by digesting the ITS PCR product with TaqI (Thermo Scientific) according to the manufacturer's instructions and separating the fragments on a 3% agarose gel. This ITC marker identified 17 variants ITC‐A to ITC‐T. Isolates were binned based on identical ITS restriction patterns (Table S1) and a representative of each group was sequenced using ITS1 (5′‐GGAAGTAAAAGTCGTAACAAGG‐3′) and ITS4 primers. Prior to sequencing, primers remaining from PCR amplification were removed by treatment with ExoSAP (Exonuclease I, Shrimp Alkaline Phosphatase; Thermo Scientific; https://www.fishersci.fi/). Assembled complete ITS regions (ITS1 – 5S rRNA – ITS2) were queried against sequences of known fungi using the Basic Local Alignment Search Tool (BLAST) at the NCBI (https://www.ncbi.nlm.nih.gov/). The *rco1 gyp7* RsaI (RGR) CAPS marker was amplified with the primers, gyp7_forward (5′‐TGTTGCTCATCATCTACAAGCG‐3′) and rco1_reverse (5′‐GTCTACTCGGTCGCCTTCTC‐3′), and the PCR product digested with the RsaI restriction endonuclease according to the manufacturer's instructions.

### Morphological characterization

*Taphrina* strains were grown at 0.2× PDA medium using streak technique on 9 cm diameter Petri dish and were cultivated at constant 23°C in a controlled environment chamber (Sanyo MLR‐350) for 3 days. Microscopic cell investigation including length, width and shape was performed on 3‐day‐old cultures. Cell images were captured by LEICA 2500 microscope with camera LEICA DFC490. Cell length and width were measured with ImageJ software (imagej.nih.gov/ij/). The 22 birch‐associated *Taphrina betulina* strains that were selected for further analysis are available from the University of Helsinki Microbial Domain Biological Resource Centre (HAMBI; https://www2.helsinki.fi/en/infrastructures/biodiversity‐collections/infrastructures/microbial‐domain‐biological‐resource‐centre‐hambi) and the German Collection of Microorganisms and Cell Cultures GmbH (DSMZ; https://www.dsmz.de/) under the accession numbers listed in Table 5.

### Indolic compound production and auxin response assay

Salkowski reagent colorimetric assay (Glickmann and Dessaux, 1995) was used to detect indolic compounds. Two different liquid media, yeast extract, peptone, dextrose (YPD) and YPD + 0.1% tryptophan, were used to cultivate 22 *T*. *betulina* strains for 5 days. Yeast cells were sedimented by centrifugation (12 000 rpm, 20°C for 5 min) and 0.5 ml culture supernatant mixed with 0.5 ml Salkowski reagent, and then *A*_530_ was measured. The experiment was performed in three independent biological replicates, each with three technical replicates.

For visualizing the activation of the plant auxin transcriptional response, *Arabidopsis thaliana* (hereafter referred to as *Arabidopsis*) Col‐0 accession plants transgenically bearing the auxin‐responsive promoter::reporter system, DR5::GUS (β‐glucuronidase) were used. Seeds were sown on 0.5× MS agar in six well plates, five plants per well, stratified in the dark at 4°C for 2 days, and transferred to a growth chamber with 12/12 h light/dark cycle with 150 μM m^−2^ irradiance at 24°C. Ten‐day‐old DR5::GUS seedlings were treated overnight with 1 ml filtered culture supernatants from 5‐day‐old *T*. *betulina* cultures. Positive controls were treated with 5 μM IAA, negative controls with filtrate from fresh, sterile medium. GUS staining solutions were prepared with 1 mM 5‐bromo‐4‐chloro‐3‐indolyl b‐d‐glucuronide dissolved in methanol, 5 mM potassium ferricyanide, and 5 mM potassium ferrocyanide in 50 mM sodium phosphate buffer (pH 7.2). For histochemical staining, seedlings were fixed with ice‐cold 90% acetone for 1 h, washed two times with ice‐cold wash solution (36 mM Na_2_HPO_4_, 14 mM NaH_2_PO_4_, pH 7.2), 30 min for each wash. Seedlings were vacuum infiltrated for 5 min and kept at room temperature in GUS staining solution. Stained seedlings were washed two times with absolute ethanol and stored in 70% ethanol.

## Supporting information


**Fig. S1.** Sampled leaves. Photos of the nine birch leaf samples (A‐I) used in this study for isolation of phyllosphere yeasts. The panel labels (A‐I) correspond to the sample names.

**Fig. S2.** Classification of the 22 *T*. *betulina* strains selected for further analysis. Classification is based on the three types of host the strains were collected from; type I, symptomatic branches from symptomatic trees; type II, asymptomatic branches from symptomatic trees; type III, asymptomatic branches from asymptomatic tree.

**Fig. S3.** PCR markers used to classify *Taphrina betulina* strains. (A) The nuclear rRNA internal transcribed spacer (ITS) TaqI cleaved amplified polymorphic sequence (CAPS) (ITC) marker based on digestion of the ITS PCR product (Containing ITS1‐5S‐ITS2 sequences) with the restriction endonuclease TaqI. (B) Schematic of new marker design. Two conserved housekeeping gene pairs were found with conserved synteny in multiple species of *Taphrina* and primers designed as depicted to allow primer binding in conserved gene regions and amplification of polymorphic intergenic regions. (C) Gene and primer names with their expected PCR products and expected intergenic region lengths. (D) Test PCR results using *T*. *betulina* and *T*. *deformans* genomic DNA as template. (E) PCR amplification results using the *gyp7*‐*rco1* primer set with a larger collection of genomic DNA templates from known birch‐associated *Taphrina* species. Genotypes with the marker using these primers (the *rco1 gyp7* RsaI (RGR) CAPS marker) are also listed. (F) *gyp7*‐*rco1* cleaved amplified polymorphic sequence (CAPS) results after PCR product digestion with the RsaI restriction endonuclease. This marker is termed the *rco1 gyp7* RsaI (RGR) CAPS marker and was tested on a collection of known birch‐associated *Taphrina* species and example strains from this study in order to illustrate the expected marker banding patterns. For estimated band sizes associated with each RGR genotype see Table S3. (G) Alignments between *T*. *betulina* strain PYCC 5889 (=CBS 119536 = NRRL T‐726; ITS accession AF492080.1), *T*. carnea strain PYCC 5890 (=NRRL T‐705; ITS accession AF492084.1), *T*. nana strain PYCC 5716 (=CBS 336.55; ITS accession AF492102) *T*. *robinsoniana* strain NRRL T‐732 (ITS accession AF492116.1), and *T*. *americana* strain PYCC 5701 (ITS accession AF492078).

**Fig. S4.** Colony and yeast cell morphology. Twenty‐two selected *Taphrina betulina* strains isolated from birch leaves. Organized by sample type, the strain numbers were; Type I – strains 25, 26, 34, 85, 31; Type II – strains 82, 219, 58, 83, 59, 68, 69, 198, 62, 199, 151, 63, 112; Type III – strains 129, 11, 19, 20.

**Fig. S5.** Activation of auxin dependent transcriptional response *in planta*. Response was monitored by the auxin, (indole acetic acid, IAA) ‐responsive DR5 promoter fused to the β‐glucuronidase (*GUS*) reporter gene. Two weeks old *in vitro* grown *Arabidopsis thaliana* seedlings transgenically bearing *DR5*::*GUS* were treated for 24 h with filtered supernatants of five‐day‐old *Taphrina betulina* cultures in YPD and YPD +0.1% Trp (tryptophan) and then stained for GUS activity, which deposited an insoluble blue coloured product in tissues where the promoter was active. Fresh sterile media (YPD and YPD + Trp) were used as negative controls and 5 μM IAA as a positive control.

**Table S1.** Yeast strain ITS CAPS analysis and identification.

**Table S2.** Full list of all strains isolated. Abbreviations used: nd, no data; np, no ITS PCR product.

**Table S3.** Banding patterns observed with the RGR1 (*Rco1 Gyp7* RsaI) CAPS (cleaved amplified polymorphic sequence) marker.

**Table S4.** Cell size of 22 *T*. *betulina* selected strains.
