# Supplementary material for: Distinct Taphrina strains from the phyllosphere of birch exhibiting a range of witches' broom disease symptoms
Source: Environ Microbiol. 2022 May 17;24(8):3549–64. doi: 10.1111/1462-2920.16037 (PMC9545635; doi:10.1111/1462-2920.16037)
Supplement: Supplementary file 1 — Fig. S1. Sampled leaves. Photos of the nine birch leaf samples (A‐I) used in this study for isolation of phyllosphere yeasts. The panel labels (A‐I) correspond to the sample names. [file EMI-24-3549-s001.pdf]

Supplemental Fig.S1

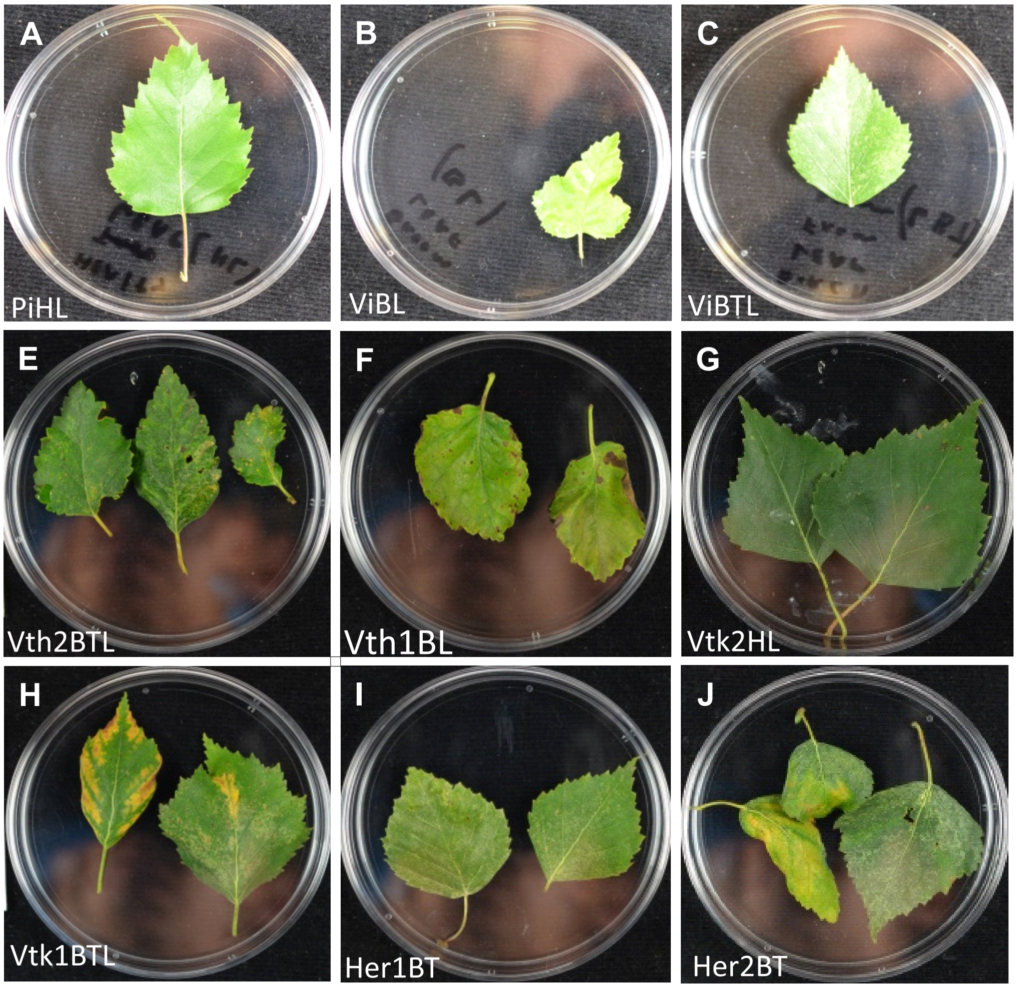

Fig. S1. Sampled leaves. Photos of the nine birch leaf samples (A-I) used in this study for isolation of phyllosphere yeasts. The panel labels (A-I) correspond to the sample names.
