# Supplementary material for: Distinct Taphrina strains from the phyllosphere of birch exhibiting a range of witches' broom disease symptoms
Source: Environ Microbiol. 2022 May 17;24(8):3549–64. doi: 10.1111/1462-2920.16037 (PMC9545635; doi:10.1111/1462-2920.16037)
Supplement: Supplementary file 2 — Fig. S2. Classification of the 22 T. betulina strains selected for further analysis. Classification is based on the three types of host the strains were collected from; type I, symptomatic branches from symptomatic trees; type II, asymptomatic branches from symptomatic trees; type III, asymptomatic branches from asymptomatic tree. [file EMI-24-3549-s005.pdf]

Supplemental Fig. 2

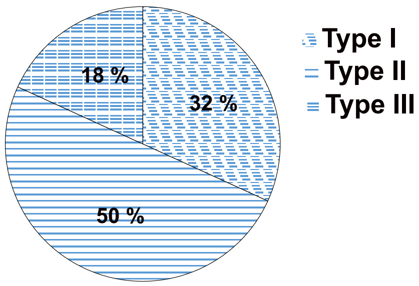

Fig. S2. Classification of the 22 *T. betulina* strains selected for further analysis. Classification is based on the three types of host the strains were collected from; type I, symptomatic branches from symptomatic trees; type II, asymptomatic branches from symptomatic trees; type III, asymptomatic branches from asymptomatic tree.
