# Supplementary material for: Distinct Taphrina strains from the phyllosphere of birch exhibiting a range of witches' broom disease symptoms
Source: Environ Microbiol. 2022 May 17;24(8):3549–64. doi: 10.1111/1462-2920.16037 (PMC9545635; doi:10.1111/1462-2920.16037)
Supplement: Supplementary file 3 — Fig. S3. PCR markers used to classify Taphrina betulina strains. (A) The nuclear rRNA internal transcribed spacer (ITS) TaqI cleaved amplified polymorphic sequence (CAPS) (ITC) marker based on digestion of the ITS PCR product (Containing ITS1‐5S‐ITS2 sequences) with the restriction endonuclease TaqI. (B) Schematic of new marker design. Two conserved housekeeping gene pairs were found with conserved synteny in multiple species of Taphrina and primers designed as depicted to allow primer binding in conserved gene regions and amplification of polymorphic intergenic regions. (C) Gene and primer names with their expected PCR products and expected intergenic region lengths. (D) Test PCR results using T. betulina and T. deformans genomic DNA as template. (E) PCR amplification results using the gyp7‐rco1 primer set with a larger collection of genomic DNA templates from known birch‐associated Taphrina species. Genotypes with the marker using these primers (the rco1 gyp7 RsaI (RGR) CAPS marker) are also listed. (F) gyp7‐rco1 cleaved amplified polymorphic sequence (CAPS) results after PCR product digestion with the RsaI restriction endonuclease. This marker is termed the rco1 gyp7 RsaI (RGR) CAPS marker and was tested on a collection of known birch‐associated Taphrina species and example strains from this study in order to illustrate the expected marker banding patterns. For estimated band sizes associated with each RGR genotype see Table S3. (G) Alignments between T. betulina strain PYCC 5889 (=CBS 119536 = NRRL T‐726; ITS accession AF492080.1), T. carnea strain PYCC 5890 (=NRRL T‐705; ITS accession AF492084.1), T. nana strain PYCC 5716 (=CBS 336.55; ITS accession AF492102) T. robinsoniana strain NRRL T‐732 (ITS accession AF492116.1), and T. americana strain PYCC 5701 (ITS accession AF492078). [file EMI-24-3549-s003.pdf]

Supplemental Fig. 3

A.

ITS TaqI CAPS marker type	Banding pattern	*Taphrina betulina type*	Closest BLAST hit
ITC-D	248,232,132, 59, 27	Variant I	Taphrina betulina strain NRRL T-726 100%
ITC-C	323, 263,59,	Variant II	Taphrina betulina strain NRRL T-726 99%

B

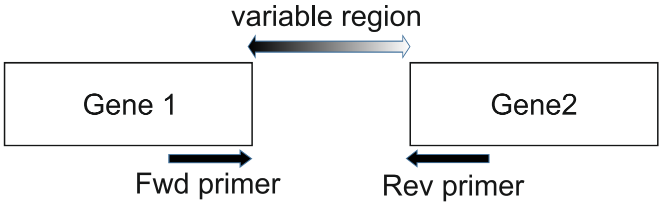

C

Forward primer	Reverse primer	Product length (bp)	variable region (bp)
sad1 fwd	rax1 rev	1250	587
gyp7 fwd	rco1 rev	1200	497

D

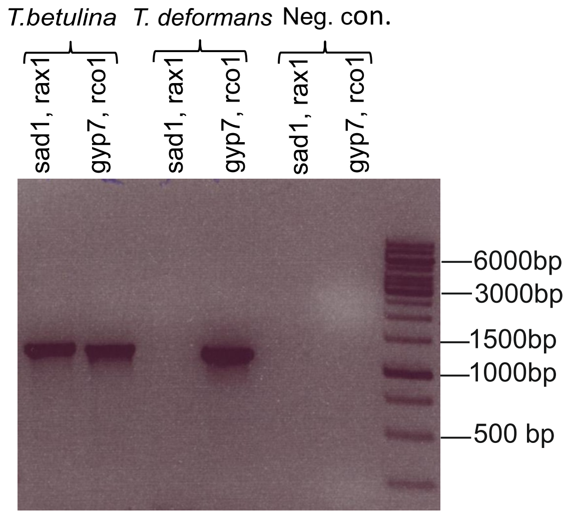

E

Species	Strain	RGR PCR	RGR type
T. betulina	PYCC 5889	+	RGR-3
T. carnea	PYCC 5890	+	RGR-1
T. deformans	PYCC 5710	+	-
T. americana	PYCC 5071		RGR-0
T. nana	PYCC 5716	+	RGR-2
T. robinsoniana	T-732	+	RGR-1

F

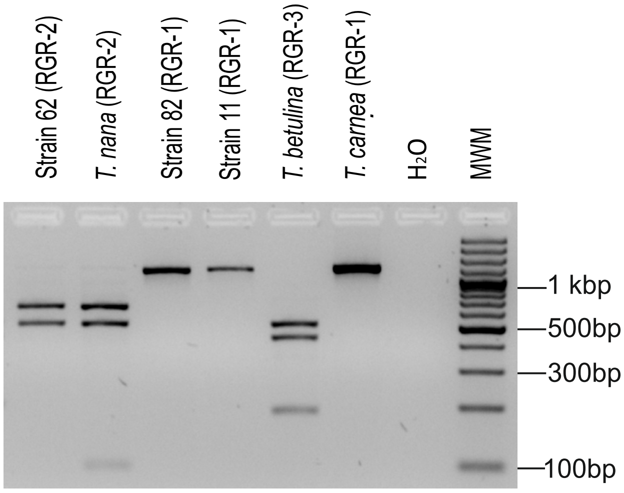

G

```
T robinsoniana    AAGGATCATTAATGAAGTCTGGGCTTCGGCCCTCTCTCTTCTACACACTTGTGAATCTAC    60
T americana       AAGGATCATTAATGAAGTCTGGGCTTCGGCCCTCTCTCTTCTACACACTTGTGAACTTAC    60
T nana            AAGGATCATTAATGAAGTCTGGGCTCCGGCCCTCTCTCTTCTACACACTTGTGAACTTAC    60
T carnea          AAGGATCATTAATGAAGTCTGGGCTCCGGCCCTCTCTCTTCTACACACTTGTGAACTTAC    60
T betulina        AAGGATCATTAATGAAGTCTGGGCTCCGGCCCTCTCTCTTCTACACACTTGTGAACTTAC    60
ITS-D             AAGGATCATTAATGAAGTCTGGGCTCCGGCCCTCTCTCTTCTACACACTTGTGAACTTAC    60
ITS-C             CGGAGGTCTTAATGAAGTCTGGGCTCCGGCCCTCTCTCTTCTACACACTTGTGAACTTAC    60
                    *     ***************** *****************************  ***
                                             ITS1

T robinsoniana    ACTGTTGCTTTGGCAGGTAGCCGGACGGACGTGAGTCTGCACGGCGAGGTCGAGAGACGC    120
T americana       ACCGTTGCTTTGGCAGGTTTCCGGAGGGGCGAAAGCTTCGAAGGTCAGGTCGAAAGGCGC    120
T nana            ACTGTTGCTTTGGCAGGTTTCCGGACGGGCGAAAGCTCTGAAGGTCAGGTCGAAAGGCGC    120
T carnea          ACTGTTGCTTTGGCAGGTTTCCGGACGGGCGAAAGCTCTGAAGGTCAGGTCGAAAGGCGC    120
T betulina        ACTGTTGCTTTGGCAGGTTTCCGGACGGGCGAAAGCTCTGAAGGTCAGGTCGAAAGGCGC    120
ITS-D             ACTGTTGCTTTGGCAGGTTTCCGGACGGGCGAAAGCTCTGAAGGTCAGGTCGAAAGGCGC    120
ITS-C             ACTGTTGCTTTGGCAGGTTTCCGGACGGGCGAAAGCTCTGAAGGTCAGGTCGGAAGGCGC    120
                  ** ***************  ***** ** **  **     * **  ******  ** ***
                                             ITS1

T robinsoniana    CTGCCAAGGACATTTATCCACCCTTTTTCAATAGTCTGATTATTGTTTTAAACAAATTAA    180
T americana       CTGCCAAGGACATTTATCCACCCTTTTTATATCGTCTGATTTTTGTTTTAAACAAATTAT    180
T nana            CTGCCAAGGACATTTACCCACCCTTTTTATATTGTCTGATTTTTGTTTTAAACAAATTAT    180
T carnea          CTGCCAAGGACATTTACCCACCCTTTTTATATTGTCTGATTTTTGTTTTAAACAAATTAT    180
T betulina        CTGCCAAGGACATTTACCCACCCTTTTTATATTGTCTGATTTTTGTTTTAAACAAATTAT    180
ITS-D             CTGCCAAGGACATTTACCCACCCTTTTTATATTGTCTGATTTTTGTTTTAAACAAATTAT    180
ITS-C             CTGCCAAGGACATTTACCCACCCTTTTTATATTGTCTGATTTTTGTTTTAAACAAATTAT    180
                  **************** ***********  **  ******** *****************
                                             ITS1

T robinsoniana    AATAAAACTTTCAACAATGGATCTCTTGGCTCTGGCATCGATGAAGAACGCAGCGAAATG    240
T americana       AATAAAACTTTCAACAATGGATCTCTTGGCTCTGGCATCGATGAAGAACGCAGCGAAATG    240
T nana            AATAAAACTTTCAACAATGGATCTCTTGGCTCTGGCATCGATGAAGAACGCAGCGAAATG    240
T carnea          AATAAAACTTTCAACAATGGATCTCTTGGCTCTGGCATCGATGAAGAACGCAGCGAAATG    240
T betulina        AATAAAACTTTCAACAATGGATCTCTTGGCTCTGGCATCGATGAAGAACGCAGCGAAATG    240
ITS-D             AATAAAACTTTCAACAATGGATCTCTTGGCTCTGGCATCGATGAAGAACGCAGCGAAATG    240
ITS-C             AATAAAACTTTCAACAATGGATCTCTTGGCTCTGGCATCGATGAAGAACGCAGCGAAATG    240
                  ************************************************************
                                            5S rRNA
```

```
T robinsoniana      CGATAAGTAATGTGAATTGCAGAATTCAGTGAATCATCGAATCTTTGAACGCACATTGCG   300
T americana         CGATAAGTAATGTGAATTGCAGAATTCAGTGAATCATCGAATCTTTGAACGCACATTGCG   300
T nana              CGATAAGTAATGTGAATTGCAGAATTCAGTGAATCATCGAATCTTTGAACGCACATTGCG   300
T carnea            CGATAAGTAATGTGAATTGCAGAATTCAGTGAATCATCGAATCTTTGAACGCACATTGCG   300
T betulina          CGATAAGTAATGTGAATTGCAGAATTCAGTGAATCATCGAATCTTTGAACGCACATTGCG   300
ITS-D               CGATAAGTAATGTGAATTGCAGAATTCAGTGAATCATCGAATCTTTGAACGCACATTGCG   300
ITS-C               CGATAAGTAATGTGAATTGCAGAATTCAGTGAATCATCGAATCTTTGAACGCACATTGCG   300
                    ************************************************************
                                              5S rRNA

T robinsoniana      CCCTCTGGTATTCCGGAGGGCATGCCTGTTTGAGTGTCATTAATCTCTCACAAAGACCTT   360
T americana         CCCTCTGGTATTCCGGAGGGCATGCCTGTTTGAGTGTCATTAATCTCTCACAAAAC-CTT   359
T nana              CCCTCTGGTATTCCGGAGGGCATGCCTGTTTGAGTGTCATTAACTTCTCACAAAAC-CTT   359
T carnea            CCCTCTGGTATTCCGGAGGGCATGCCTGTTTGAGTGTCATTAACTTCTCACAAAAC-CTT   359
T betulina          CCCTCTGGTATTCCGGAGGGCATGCCTGTTTGAGTGTCATTAACTTCTCACAAAAC-CTT   359
ITS-D               CCCTCTGGTATTCCGGAGGGCATGCCTGTTTGAGTGTCATTAACTTCTCACAAAAC-CTT   359
ITS-C               CCCTCTGGTATTCCGGAGGGCATGCCTGTTTGAGTGTCATTAACTTCTCACAAAAC-CTT   359
                    *******************************************  *********   ***
                                      5S rRNA                          ITS2

T robinsoniana      TTGGTCTCTGGTGAAGTTGGGATCTGCGTACCTTTGTGGAACGCTGTCCCCAAATAGATT   420
T americana         TTGGTTTCTGTCGATGTTGGGAGCTGCGACCCCTCGTGGGACGCTCTCCTTAAATGTATT   419
T nana              TTGGTTTCTGTTGATGTTGGGAACTGCGACCCCTCGTGGGACGCTTTCCTCAAATGCATT   419
T carnea            TTGGTTTCTGTTGATGTTGGGAACTGCGACCCCTCGTGGGACGCTTTCCTCAAATGCATT   419
T betulina          TTGGTTTCTGTTGATGTTGGGAACTGCGACCCCTCGTGGGACGCTTTCCTCAAATGCATT   419
ITS-D               TTGGTTTCTGTTGATGTTGGGAACTGCGACCCCTCGTGGGACGCTTTCCTCAAATGCATT   419
ITS-C               TTGGTTTCTGTTGATGTTGGGAACTGCGACCCCTCGTGGGACGCTTTCCTCAAATGCATT   419
                    ***** ****  ** ******* *****  ** * **** ***** ***  ****  ***
                                               ITS2

T robinsoniana      GGTGCGGCCCGCCGCCC-GGTACACAACGTTCTAGGTTCGTCCAACTCGTTGATCAACCG   479
T americana         GGTGCGGCCCGCCGCCCGGTTACACAACGTTCTAGGTTCGTCCAACTCGTTGCTCAGCCG   479
T nana              GGTGCGGCCCGCCGCCCGGTAACACAACGTTCTAGGTTCGTCCAACTCGTTGCTCGC-CG   478
T carnea            GGTGCGGCCCGCCGCCCGGTAACACAACGTTCTAGGTTCGTCCAACTCGTTGCTCGC-CG   478
T betulina          GGTGCGGCCCGCCGCCCGGTAACACAACGTTCTAGGTTCGTCCAACTCGTTGCTCGC-CG   478
ITS-D               GGTGCGGCCCGCCGCCCGGTAACACAACGTTCTAGGTTCGTCCAACTCGTTGCTCGC-CG   478
ITS-C               GGTGCGGCCCGCCGCCCGGTAACACAACGTTCTAGGTTCGTCCAACTCGTTGCTCGC-CG   478
                    ***************** *  ******************************* **   **
                                               ITS2
```

```
T robinsoniana      GTGCATATATGGTGCTGCACCTAAAGCCCGCCTTGTGCCTTGTGCTCTTGGCTCTTTT-A    538
T americana         GTGCAATCTTGGTGCTGCACCTAAAGCCCGCCTCGTGCCTTGTGCTCTTGGCTAACTTCA    539
T nana              GTGCAATCTTGGTGCTGCACCTTAAGCCCGCCCTGTGCCTTGTGCACTTGGCTAACTTCA    538
T carnea            GTGCAATCTTGGTGCTGCACCTTAAGCCCGCCCTGTGCCTTGTGCACTTGGCTAACTTCA    538
T betulina          GTGCAATCTTGGTGCTGCACCTTAAGCCCGCCCTGTGCCTTGTGCACTTGGCTAACTTCA    538
ITS-D               GTGCAATCTTGGTGCTGCACCTTAAGCCCGCCCTGTGCCTTGTGCACTTGGCTAACTTCA    538
ITS-C               GTGCAATCTTGGTGCTGCACCTTAAGCCCGCCCTGTGCCTTGTGCACTTGGCTAACTTCA    538
                    *****    ************* *********  *********** *******   ** *
                                             ITS2

T robinsoniana      TCTATTGACCTCAGATCAGGTAGGAATACGCGCTGAACTTAAGC    582
T americana         T-TATTGACCTCAGATCAGGTAGGAATACGCGCTGAACTTAAGC    582
T nana              TTTATTGACCTCAGATCAGGTAGGAATACGCGCTGAACTTAAGC    582
T carnea            TTTATTGACCTCAGATCAGGTAGGAATACGCGCTGAACTTAAGC    582
T betulina          TTTATTGACCTCAGATCAGGTAGGAATACGCGCTGAACTTAAGC    582
ITS-D               TTTATTGACCTCAGATCAGGTAGGAATACGCGCTGAACTTAAGC    582
ITS-C               TTTATTGACCTCAGATCAGGTAGGAATACGCGCTGAACTTAAGC    582
                    * ******************************************
```

Fig. S3. PCR markers used to classify *Taphrina betulina* strains. (A) The nuclear rRNA internal transcribed spacer (ITS) TaqI cleaved amplified polymorphic sequence (CAPS) (ITC) marker based on digestion of the ITS PCR product (Containing ITS1-5S-ITS2 sequences) with the restriction endonuclease TaqI. **(B)** Schematic of new marker design. Two conserved housekeeping gene pairs were found with conserved synteny in multiple species of *Taphrina* and primers designed as depicted to allow primer binding in conserved gene regions and amplification of polymorphic intergenic regions. **(C)** Gene and primer names with their expected PCR products and expected intergenic region lengths. **(D)** Test PCR results using *T. betulina* and *T. deformans* genomic DNA as template. **(E)** PCR amplification results using the *gyp7-rco1* primer set with a larger collection of genomic DNA templates from known birch-associated*Taphrina* species. Genotypes with the marker using these primers (the *rco1 gyp7* RsaI (RGR) CAPS marker) are also listed. **(F)** *gyp7-rco1* cleaved amplified polymorphic sequence (CAPS) results after PCR product digestion with the RsaI restriction endonuclease. This marker is termed the *rco1 gyp7* RsaI (RGR) CAPS marker and was tested on a collection of known birch-associated *Taphrina* species and example strains from this study in order to illustrate the expected marker banding patterns. For estimated band sizes associated with each RGR genotype see Table S3. **(G)** Alignments between *T. betulina* strain PYCC 5889 (=CBS 119536=NRRL T-726; ITS accession AF492080.1), *T.* carnea strain PYCC 5890 (=NRRL T-705; ITS accession AF492084.1), *T.* nana strain PYCC 5716 (=CBS 336.55; ITS accession AF492102) *T. robinsoniana* strain NRRL T-732 (ITS accession AF492116.1), and *T. americana* strain PYCC 5701 (ITS accession AF492078).
