# Supplementary material for: Distinct Taphrina strains from the phyllosphere of birch exhibiting a range of witches' broom disease symptoms
Source: Environ Microbiol. 2022 May 17;24(8):3549–64. doi: 10.1111/1462-2920.16037 (PMC9545635; doi:10.1111/1462-2920.16037)
Supplement: Supplementary file 4 — Fig. S4. Colony and yeast cell morphology. Twenty‐two selected Taphrina betulina strains isolated from birch leaves. Organized by sample type, the strain numbers were; Type I – strains 25, 26, 34, 85, 31; Type II – strains 82, 219, 58, 83, 59, 68, 69, 198, 62, 199, 151, 63, 112; Type III – strains 129, 11, 19, 20. [file EMI-24-3549-s007.pdf]

Supplemental Fig. 4.

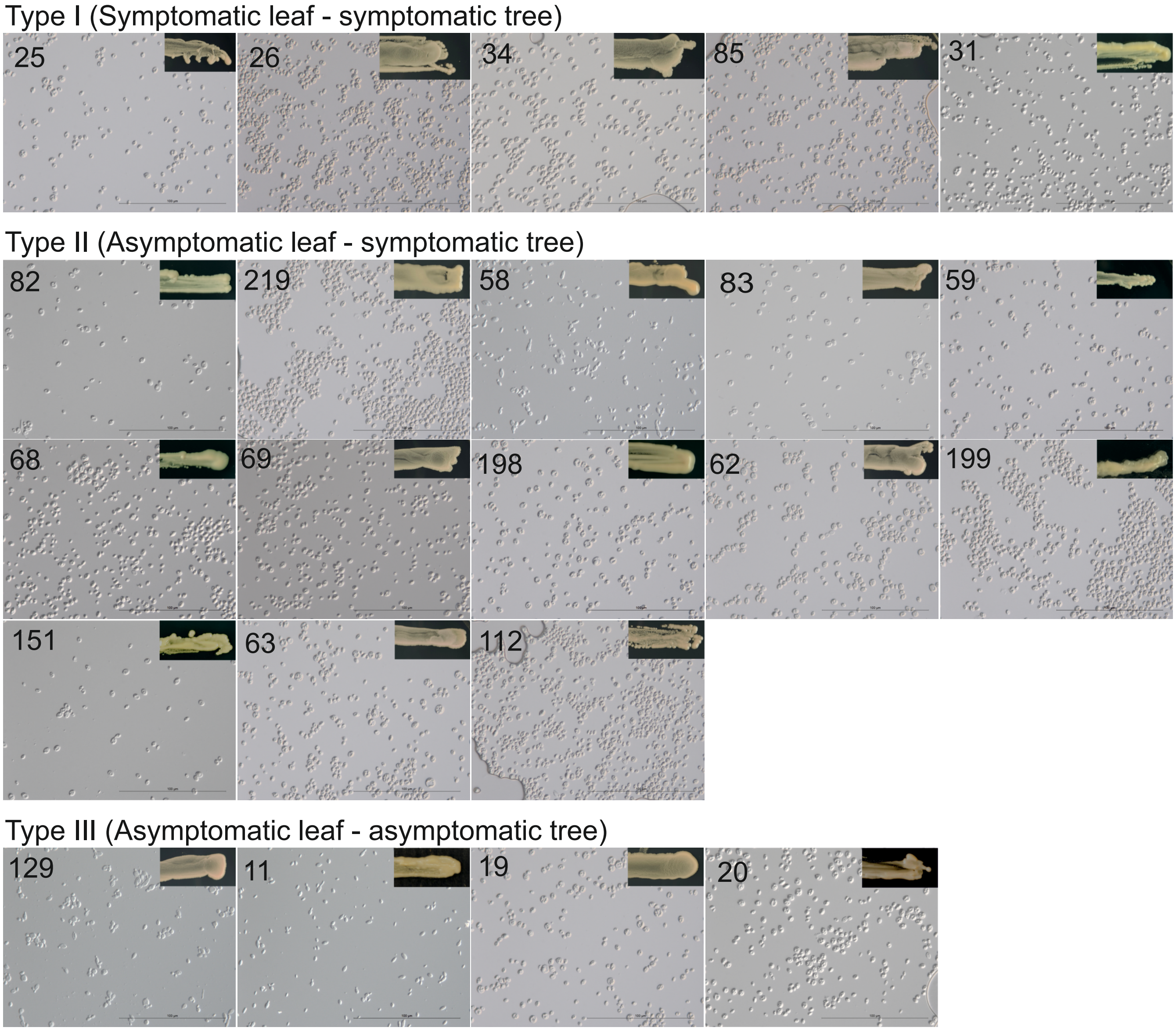

Fig. S4. Colony and yeast cell morphology. Twenty-two selected *Taphrina betulina* strains isolated from birch leaves. Organized by sample type, the strain numbers were; Type I – strains 25, 26, 34, 85, 31; Type II – strains 82, 219, 58, 83, 59, 68, 69, 198, 62, 199, 151, 63, 112; Type III - strains 129, 11, 19, 20.
