# Supplementary material for: Distinct Taphrina strains from the phyllosphere of birch exhibiting a range of witches' broom disease symptoms
Source: Environ Microbiol. 2022 May 17;24(8):3549–64. doi: 10.1111/1462-2920.16037 (PMC9545635; doi:10.1111/1462-2920.16037)
Supplement: Supplementary file 5 — Fig. S5. Activation of auxin dependent transcriptional response in planta. Response was monitored by the auxin, (indole acetic acid, IAA) ‐responsive DR5 promoter fused to the β‐glucuronidase (GUS) reporter gene. Two weeks old in vitro grown Arabidopsis thaliana seedlings transgenically bearing DR5::GUS were treated for 24 h with filtered supernatants of five‐day‐old Taphrina betulina cultures in YPD and YPD +0.1% Trp (tryptophan) and then stained for GUS activity, which deposited an insoluble blue coloured product in tissues where the promoter was active. Fresh sterile media (YPD and YPD + Trp) were used as negative controls and 5 μM IAA as a positive control. [file EMI-24-3549-s009.pdf]

Supplemental Fig. 5

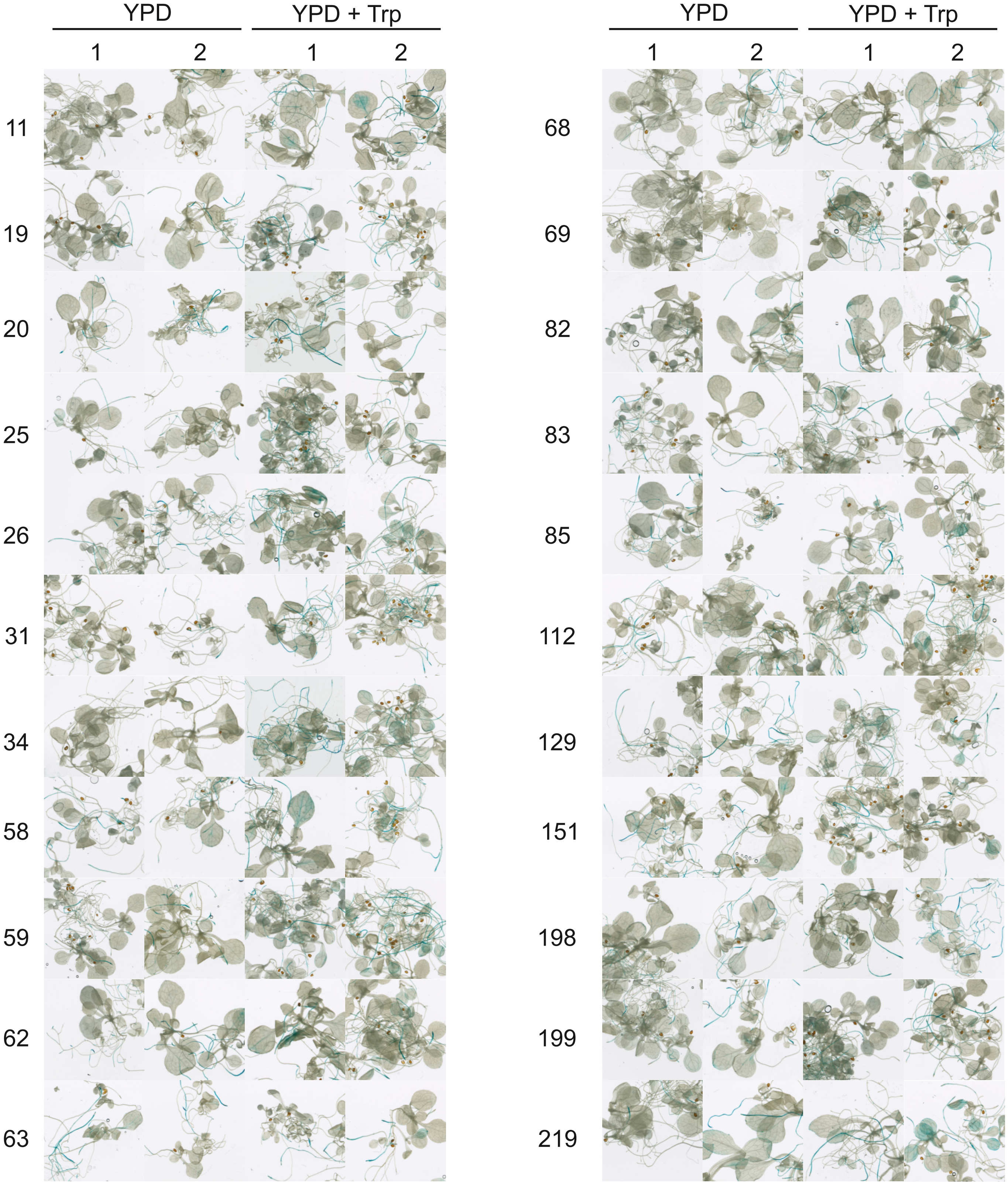

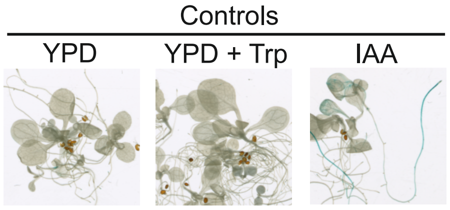

Fig. S5. Activation of auxin dependent transcriptional response *in planta.* Response was monitored by the auxin, (indole acetic acid, IAA) -responsive DR5 promoter fused to the β-glucuronidase (*GUS*) reporter gene. Two weeks old *in vitro* grown *Arabidopsis thaliana* seedlings transgenically bearing *DR5::GUS* were treated for 24 hours with filtered supernatants of five-day-old *Taphrina betulina* cultures in YPD and YPD +0.1% Trp (tryptophan) and then stained for GUS activity, which deposited an insoluble blue coloured product in tissues where the promoter was active. Fresh sterile media (YPD and YPD + Trp) were used as negative controls and 5 μM IAA as a positive control.
