# Supplementary material for: Distinct Taphrina strains from the phyllosphere of birch exhibiting a range of witches' broom disease symptoms
Source: Environ Microbiol. 2022 May 17;24(8):3549–64. doi: 10.1111/1462-2920.16037 (PMC9545635; doi:10.1111/1462-2920.16037)
Supplement: Supplementary file 6 — Table S1. Yeast strain ITS CAPS analysis and identification. [file EMI-24-3549-s004.pdf]

Supplemental Table S1: Yeast strain ITS CAPS analysis and identification

A) ITC [Internal transcribed spacer (ITS) Taq1 cleaved amplified polymorphic sequence (CAPS)] analysis

ITC Type	Number Isolates	ITS Taq I Banding Pattern	Taxon Name	Best BLAST Hit	% Identity
A	42	197, 171, 160, 59,45,27	*Microstroma sp.*	*Microstroma album*	97.9%
C	14	323, 263,59	*Taphrina betulina* Variant II	*Taphrina betulina* (NRRL T-726)	99.5 %
D	43	248,232,132, 59, 27	*Taphrina betulina* Variant I	*Taphrina betulina* (NRRL T-726)	100%
E	25	188, 150, 131, 59, 53,53	*Elsinoe* sp.	*Elsinoe eucalyptorum*	96.7 %
F	35	232, 132, 59,39, 27	*Pseudomicrostroma* sp.	*Pseudomicrostroma glucosiphilum*	95.5 %
H	1	327, 210,59	*Cystobasidium ritchiei*	*Cystobasidium ritchiei*	99.0 %
I	4	359,222, 59	*Filobasidium wieringae*	*Filobasidium wieringae*	98.5 %
L	2	185, 151, 148, 100,59, 20	*Itersonilia pannonica*	*Itersonilia pannonica*	99.7 %
M	4	265, 228,59	*Vishniacozyma tephrensis*	*Vishniacozyma tephrensis*	99.4 %
N	1	232,147,139,107,76,59	*Kuraishia capsulata*	*Kuraishia capsulata*	99.6 %
O	1	316,300, 59	*Nakazawaea holstii*	*Nakazawaea holstii*	100 %
S	1	296, 220, 29	Novel Tremellaceae	*Tremella* sp. strain KBP:Y-6427	99.2 %
NP [a]	33	-	-	-	-
UI [b]	18	-	-	-	-

[a] NP, no ITS PCR product, confirmed bacterial and removed from further analysis. [b] UI, unidentified due to poor ITS amplification or multiple ITS bands and removed from further analysis.

B) ITS sequences of isolates

>ITC_A
TTTTGGGTGACTGCAGACGCTCATTAATGATTCTAGGGATCTTCCATTCTCATGGATTCCCGAACCCTCACCTTTATCCAATTACACCTGTGCACTGTTGGTTAGGTGTCTCCAATTTAGGAGATTCATCTGCCAACGCC
AAATTTAACAAACCCTGAGTTTATTGAATGTGAATTAACTATGCCTTTAGTTGGGCAGAAAATTAATACAACTTTCGACAACGGATCTCTTGGTTCTCCCATCGATGAAGAACGCAGCGAAATGCGATAAGTAATGTGAAT
TGCAGAATTCAGTGAATCATCGAATCTTTGAACGCACCTTGCGCTCCTGGGTATTCCCAGGAGCATGCCTGTTTGAGTGTCATGAATCCCTCAAATCCCAATGTTTTGCAAAACTCATTGCTTGGATTTGGTCATGGGC
CGACTTTGCTACTTTATTGTTAGCTGGCCTTAAAGACATTAGCTTGGACTTTCGAACCTTTTCGGAAGGGTCTCCATATGATGTGATAAATTTACGTCGATGGGCTAATGACTCTACTGTTGTTAATGGTTTTGAAAACGA
ATAGTCTAGCTTTCTAACCCGGCGAAGAGTTCCATCTCTGACCCACTTTTCATTAACTCTGGCCTCAAATCAGGTAGGACTACCCGCTGAACTTAAGCATATCAATAAGCGGAGGAAAAGAAACTAACAAGGATTCCCC
TAGTAACGGCGAGTGAAGCGGGAAGAGCTCAAATTTGAAAGCTGGCGCCTTCGGCGTCCGCGTTGTAATCTCGAGAAGTGTTTTCCGTGCTGGACCATGTACAAGTTCCTTGGAATAGACGTCATAGAGGGTGAAAAT
CCCGTCCTTGACATGGACGCCCAGTGCTTTGTGATACGCTCTCCACGAGTCGAGTTGTTTGGGAATGCAGCTCAAATGGGTGGTAAATTCCATCTAAAGCTAAATATTGGGGAGAGACCGATAGCGAACAAGTACCGT
GAGGGAAAGATGAAAAGCACTTTGGAAAGAGAGTTAAACAGTACGTGAAATTGTCGAAAGGGAGCGCTTGAGTTAGACATGCCTGCTGGATTCAGCCTTGCTTTTTGCTTGTGTATTTTCCGTGAGCAGCCAGCATCA
GTTTTGTCTGTCGGATAAGGTAGGAGGAACGTAGCCCCCCCTCGGGGGGTGGTTATAGCCCTCTTACTGGAATACCGGCGGATGGGGACTGA
>ITC_C
CTGAAGTGACTGCGGAGGTCTTAATGAAGTCTGGGCTCCGGCCCTCTCTCTTCTACACACTTGTGAACTTACACTGTTGCTTTGGCAGGTTTCCGGACGGGCGAAAGCTCTGAAGGTCAGGTCGGAAGGCGCCTGCC
AAGGACATTTACCCACCCTTTTTATATTGTCTGATTTTTGTTTTAAACAAATTATAATAAAACTTTCAACAATGGATCTCTTGGCTCTGGCATCGATGAAGAACGCAGCGAAATGCGATAAGTAATGTGAATTGCAGAATTC
AGTGAATCATCGAATCTTTGAACGCACATTGCGCCCTCTGGTATTCCGGAGGGCATGCCTGTTTGAGTGTCATTAACTTCTCACAAAACCTTTTGGTTTCTGTTGATGTTGGGAACTGCGACCCCTCGTGGGACGCTTT

CCTCAAATGCATTGGTGCGGCCCGCCGCCCGGTAACACAACGTTCTAGGTTCGTCCAACTCGTTGCTCGCCGGTGCAATCTTGGTGCTGCACCTTAAGCCCGCCCTGTGCCTTGTGCACTTGGCTAACTTCATTTATT
GACCTCAGATCAGGTAGGAATACGCGCTGAACTTAAGCATATCAATAAGCGCAGGAAAAGAAACTAACTAGGATGCCTTCAGTAACGGCGAGCGAAGCAGGCATAGCTCAAATTTGTAATCTGGCGCCTTTGGCGTCC
GAGTTGTATTTTCTAGAAGCGAGTTCGGCGACAGCTCCTACCCAAGTCCGTTGGAACGCGGCGTCATGGAGGGTGAGAATCCCGTGAATGGTAGGATGTCTGCTGCTATGTGAACCGCCTTCGAAGAGTCGAGTTGT
TTGGGAATGCAGCTCTAAATGGGAGGTAAATTTCTTCTAAAGCTAAATATTGGCCAGAGACCGATAGCGAACAAGTAGAGTGATCGAAAGATGAAAAGAACTTTGAAAAGAGAGTTAAACAGTACGTGAAATTGTTGAA
AGGGAAGCGCTTGAGATCAGACTTCCTGTCTGGACTGTCCTTGCTTTTGCTTGGGTCAACAGTCCAGCGTGAGGCCAGCATCAGTTTTGACGGCGGATAAAGGCCTTGGAAGTAGCTCTCTCGGGAGTGTTATAGCC
CAGGTGTAATACGGCCAGTTGGACTGAGACGCGCTCTGCCTAGATGCTGGCGTATGTCTTAAGCGACCGTCTTTGGAACCACGGAACCAGGGAGTCTTAAACATCTGTTG
>ITC_D
AAGGATCATTAATGAAGTCTGGGCTCCGGCCCTCTCTCTTCTACACACTTGTGAACTTACACTGTTGCTTTGGCAGGTTTCCGGACGGGCGAAAGCTCTGAAGGTCAGGTCGAAAGGCGCCTGCCAAGGACATTTACC
CACCCTTTTTATATTGTCTGATTTTTGTTTTAAACAAATTATAATAAAACTTTCAACAATGGATCTCTTGGCTCTGGCATCGATGAAGAACGCAGCGAAATGCGATAAGTAATGTGAATTGCAGAATTCAGTGAATCATCGA
ATCTTTGAACGCACATTGCGCCCTCTGGTATTCCGGAGGGCATGCCTGTTTGAGTGTCATTAACTTCTCACAAAACCTTTTGGTTTCTGTTGATGTTGGGAACTGCGACCCCTCGTGGGACGCTTTCCTCAAATGCATT
GGTGCGGCCCGCCGCCCGGTAACACAACGTTCTAGGTTCGTCCAACTCGTTGCTCGCCGGTGCAATCTTGGTGCTGCACCTTAAGCCCGCCCTGTGCCTTGTGCACTTGGCTAACTTCATTTATTGACCTCAGATCAG
GTAGGAATACGCGCTGAACTTAAGC
>ITC_E
GGATCATTAAGAGTTAGGGTCCCTAGTGGGCCCGAACCTCCAACCCTTTGTTGTCGAACAAAAAATCGTTGCTTTGGCGGGACCGTCCCCCCCTTCACGGGGGGAGGGCTGCCGGGCTCTTAACCGAGCCGGGTAT
GCGCCCGCCAGAGTCAAACTCCAACTCTTCTCTTGAACAGTACAGTCTGAGTAAAAACTTTTAATTAAATTAAAACTTTCAACAACGGATCTCTTGGTTCTGGCATCGATGAAGAACGCAGCGAAATGCGATAAGTAATG
TGAATTGCAGAATTCAGTGAATCATCGAATCTTTGAACGCACATTGCGCCCCTTGGTATTCCGGGGGGCATGCCTGTTCGAGCGTCATTACACCAATCAAGCACCGCTTGGTATTAGGTCTCTTCGCCTCCCCTGCCCT
CACCGGCGGGAGGCGGGCCTCAAACACTTCGGCGGGGCCTCTTGGGCTTTGGGCGTAGCAGAATTTATCTAAAACGTCCTTGGGTCTCTCGAGTCTCCTGCCGCTTGAAACCGAATCGGAACACCCCGTGCGAGCG
GGAATTCCCCTTCACTTTTTCAAAGGTTGACCTCGGATCAGGTAGGAATACCCGCTGAACTTAAGCATATCAATAAGCGGAGGAAAAGAAACCAACAGGGATTGCCTCAGTAACGGCGAGTGAAGCGGCAACAGCTCA
AATTTGAAATCTGGCCCTTTCAGGGTCCGAGTTGTAATTTGTAGAGGATGCTTTTGGGCAGCCACCGGTCTAAGTTCCTTGGAACAGGACGTCACAGAGGGTGAGAATCCCGTACGTGACCGGCAGGCACCCTCCGT
AAAGCTCCTTCGACGAGTCGGGTTGTTTGGGAATGCAGCCCTAAATTGGAGGTAAATTTCTTCTAAAGCTAAATACCGGCCAGAGACCGATAGCGCACAAGTAGAGTGATCGAAAGATGAAAAGCACTTTGGAAAGAG
AGTTAAAAAGCACGTGAAATTGTTGAAAGGAAGCGCTTGCAATCAGTCTCGACGGCG
>ITC_F
CTCTGGTACTGCGAGATCATTAGTGATTTTGTATGGGTTTTCCACTCTGTGGTAACCCTCACTACAATACACCAACATCCATATACACCTGTGCACCGTTGGCTAGATGTGGCTCATTCGTGAGTCTTCGTCTGCTAACA
ACAATTTATAAACTCTGAGTTATAATGAATGTTAATTGTATCTTGCCATTTGGCAGAAACTAAATACAACTTTCGACAACGGATCTCTTGGTTCTCCCATCGATGAAGAACGCAGCGAATTGCGATAAGTAATGTGAATTG
CAGAATTCAGTGAATCATCGAATCTTTGAACGCACCTTGCGCTCCCTGGTATTCCTAGGAGCATGCCTGTTTGAGTGTCATGAATCCCTCAAATCCCAATGTTTTTTTAAAAAGAAGCGTTGCTTGGATTTGGTTGTGGG
CCCTTGCAGTCTTTCACGAGTCTGCTGGCCTTAAAGATATTAGCTGGACCTCTCTAATCTCTAAGCAGAGTACTTCCATTTGATGTAATAAAATTGCATTGGTGGAAGCTCGACTTGGTACCTAGAGGACGTCTGCTTCT
TAACCCGGCGCGAGTGGCAACACTCAACCCTCTATTTCATAACTCTGGCCTCAAATCAGGTAGGACTACCCGCTGAACTTAAGCATATCAATAAGCGGAGGAAAAGAAACTAACAAGGATTCCCCTAGTAACGGCGAG
TGAAGCGGGAAGAGCTCAAATTTGAAAGCTGGTACCTTCGGTGCCCGCGTTGTAATCTCGAGAAGTGTTTTCCGTGCTGGACCATGTACAAGTTCCTTGGAATAGGACGTCATAGAGGGTGAAAATCCCGTACTTGAC
ATGGACGCCCAGTGCTTTGTGATACACTCTCCACGAGTCGAGTTGTTTGGGAATGCAGCTCAAAATGGGTGGTAAATTCCATCTAAGCTAAATATTGAGGAGAGACCGATAGCGAACAAGTACCGTGAGGGAAAGATG
AAAAGCACTTTGGAAAGAGAGTTAGACAGTACGTGAAATTGTCTAAAGGGAAGCGCTTGAAGTAGACATGCCTATCAGGATTCAGCCTGCTTTGCTCGTGTATTCCTGGTAGCAGCAGCATCAGTTTTGTCTGTCGATA
GGTAGATGAATGTGACCCCTCGGGTGTATAGCATTTACTGATACGCGATGACTGAAGACTGCATGCGCCTTATGGC GGCTCGCACCATCGCACTAGGATGCTGGCGTACTGGCA
>ITC_H
GCGTAAAGATGGCGAGTACTGCGGAGGACATTAATGAATTTTAGGACTCTCTTTTTAGAGGTCCGACCCTTTCATTTCCATACACTGTGCACACACTTCTTTTCACACATTTTAACACTATAGTATAAGAATGTAACAGTC
TCTTTATTGAGCATAAATAAAAATAAAACTTTCAGCAACGGATCTCTTGGCTCTCGCATCGATGAAGAACGCAGCGAATTGCGATAAGTAATGTGAATTGCAGAATTCAGTGAATCATCGAATCTTTGAACGCACCTTGC
ACTCTTTGGTATTCCGAAGAGTATGTCTGTTTGAGTGTCATGAAACTCTCAACCCCCCTATTTTGTAATGAGATGGGCGTGGGCTTGGATTATGGCTGTTTGTCGGCGTAAATGCCGGCTCAGCTGAAATATACGAGCA
ACCCATTTGAAATAAACGGTTTGACTTGGCGTAATAATTATTTCGCTGAGGACGTTTTCTTCAAAAGTAAGAGGTGCTTCTAATGCGCTTTTATAGCACTTTATATTTTAGACCTCAAATCAGTCAGGACTACCCGCTGAA
CTTAAGCATATCAATAAGCGGAGGAAAAGAAACTAACAAGGATTCCCCTAGTAACGGCGAGTGAAGTGGGAAAAGCTCAACTTTGAAATCTGGTACTTTCAGTATCCGAATTGTAGTCTCAAGAAATGTTTTCTGTGCC
GGTCCGTGTATGAGTCTGTTGGAACACAGCGTCATAGAGGGTGACAACCCCGTTCATGACACGGATACCGGTGCTTTGTGATACATTCTCGAAGAGTCGAGTTGTTTGGGAATGCAGCTCAAATTGGGTGGTAAATTC
CATCTAAAGCTAAATATTGGCGAGAGACCGATAGCAAACAAGTACCGTGAGGGAAAGATCGAAAAGCACTTTGGAAAGAGAGTTAACAGTACGTGAATTTGTTGGAAAGGAAAACGTATGAAGTCAGACGTGCGTGAT
GGCGGTTCAGCTCTGGTTCCCCAGAAATGTCTTCCGTTAT
>ITC_I
CTATAGGGTGACTGCGGAGGATCATTAATGAAATGTGTTGCCGGGGCCCATAATCCCGGCACTAACCTTCTTATCCATAACACCTGTGCACTGTTGGATGCTTGCATCCACTTTTATACTAAACAATTTGTAACAAATGT
AGTCTTATTATAATTAATAAAACTTTTAACAACGGATCTCTTGGCTCTCGCATCGATGAAGAACGCAGCGAAATGCGATAAGTAATGTGAATTGCAGAATTCAGTGAATCATCGAATCTTTGAACGCACCTTGCGCTCCTT
GGTATTCCGAGGAGCATGCCTGTTTGAGTGTCATAAACCTCTCAAACCCAAGTTTTGGATTTATCCTTGCTTGAGTTTGGATTTGGGTGTTTGCCAGTGATGAACTGACTCACCTTAAAAGTATTAGCTAGATCTGTCTTT
GACTGGTTTGACTTGGCATAATAAGTATTTTGCTAAGGACATCTTCGGATGGCCAGGACTTGACTTTTGTCTGCTTACTAAACCTTACTTTAAGTGCATCTCTGGTGTTACTTATAGTATTACTTTGACATATGGCCTCAA
ATCAGGTAGGACTACCCGCTGAACTTAAGCATATCAATAAGCGGAGGAAAAGAAACTAACAAGGATTCCCCTAGTAACGGCGAGTGAAGCGGGAAGAGCTCAAATTTGAAATCTGGTGGCCTCAGGTCATCCGAGTTG
TAATCTATAGAAACGTTTTCCGTGCTGGCTCATGTACAAGTCCCTTGGAACAGGGCGTCATAGAGGGTGAGAATCCCGTCCTTGACATGAACTACCAGTGCTCTGTGATACGTTTTCAACGAGTCGAGTTGTTTGGGAA
TGCAGCTCAAAATGGGTGGTAAATTCCATCTAAAGCTAAATATTGGCGAGAGACCGATAGCGAACAAGTACCGTGAGGGAAAGATGAAAAGCACTTTGGAAAGAGAGTTAAACAGTATGTGAAATTGTTAAAAGGGAAA
CGATTGAGTCAGTCGTGCTCTTTGGATTCAGCCGGTTCTGCCGGTGTACTTCCATTGAGTGGGGTCAACATCAGTTTTGATCGCTGGATAAGGCAGGAGATGTAGCACTCGGTGACTTATAGCTTTCTGTCACATACAG
TGGTGGGACTGAGACGCAGCATGCTTTATGCCGGATCGTCACGTACATGCTTAGGATGTTGACGTAATGGCTTTAACGACCCGTCTTGGAGC
>ITC_L
ATCACTAGTGATACATCGGGGAGCGTCTTAACTGACCTCCCCAACCCTTCACAATCCACATACACCTGTGCACTGTTTTGGCTTTTGAGCAATCGAAAGCCAATCATTTTATACAAACCCCTAGTTTAATGAATGTAACC
GTTTTATATAAACAAAATAAAACTTTCAACAACGGATCTCTTGGTTCTCGCATCGATGAAGAACGCAGCGAAATGCGATAAGTAATGTGAATTGCAGAATTCAGTGAATCATCGAATCTTTGAACGCACCTTGCGCTCCTT
GGTATTCCGAGGAGCATGCCTGTTTGAGTGTCATGAAACTCTCAACCTCTGACTTTCTTAATCGTCGGTCAGCGGCTTGGATGTGGGCGCTGCCGGTCTTAATTGATCGGCTCGCCTGAAAAACATTAGCGAATCCCT
TCTGTAATCGGTTCCACTCGACGTGATAAGTATTTCGTCGAGGACATCTTTATTGATGGCCGAGATAAAGCTGGTTTAAGATCTGCTTCTAACTCTTTCGTTGCCTTCTGGTAAAGAAAACCCCTCATGATCTGGCCTCA
AATCAGGTAGGACTACCCGCTGAACTTAAGCATATCAATAAGCGGAGGAAAAGAAACTAACAAGGATTCCCCTAGTAACGGCGAGTGAAGCGGGATGAGCTCAAATTTAAAATCTGGCTGCCTCAGGTGGTCCGAGTT

GTAGTCTCGAGAAGTGTTTTCCGCGTTGGCCTGTGTACAAGTCCTTTGGAATAAGGCGTCATAGAGGGTGAGAATCCCGTCCTTGACACAGATCCCCAATGCTTTGTGATGCGCTCTCAAAGAGTCGAGTTGTTTGGG
AATGCAGCTCAAAATGGGTGGTAAATTCCATCTAAAGCTAAATATTGGCGAGAGACCGATAGCGA
>ITC_M
CTAAAGGGGGACTGCGGAGATCATTAATAATGCCCTCTGGCTTCGGTCAGCTGGGTTCAATGTGTGTCATCTCTTCGGAGATGGCCATCCACACACACCGTGAACTGTGACTTCGGTCATCACAAACTTTTAGTAATGA
ATGTAAAAATCATAACAAAATAAAACTTTTAACAACGGATCTCTTGGCTCTCGCATCGATGAAGAACGCAGCGAAATGCGATAAGTAATGTGAATTGCAGAATTCAGTGAATCATCGAATCTTTGAACGCACCTTGCGCC
CTTTGGTATTCCGAAGGGCATGCCTGTTTGAGTGTCATGAAACCTCACTCCACTTGGGTTTTTGCCTGAGTGGTAGTGTATTGGGTGTTGCCTTGCCAAAGGCTCGCCTTAAAAGAATAAGCACCTTGGATGTAATACG
TTTCATCCTTTCGGGTGGCTAATAACCCCACATAACTCATGATCTGGCCTCAAATCAGGTAGGGCTACCCGCTGAACTTAAGCATATCAATAAGCGGAGGAAAAGAAACTAACTAGGATTCCCTTAGTAACGGCGAGTG
AACCGGGATGAGCTCAAATTTGAAATCTGGCGTCTTTCAGGCGTCCGAGTTGTAATCTATAGAGGCGTTTTCCGCGCCGGACCGCGTCCAAGTCTCTTGGAATAGAGTATCAAAGAGGGTGACAATCCCGTACTTGAC
GCGACAACCGGTGCTCTGTGATACGTTCTCAACGAGTCGAGTTGTTTGGGAATGCAGCTCTAAATGGGTGGTAAATTCCATCTAAGGCTAAATATTGGCGAGAGACCGATAGCGAACAAGTACCGTGAGGGAAAGATG
AAAAGCACTTTGGAAAGAGAGTTAAACAGCACGTGAAATTGTTAAAAGGGAAACGATTGAAGTCAGTCGTGTGAAAGGTATTCAGCCGTCTCTGGCGGTGTATTTGCCTTTCACGGGTCAACATCAGTTTAGTCCGGTA
GAAAAAGCTGGAGGAAGGTGGCACCCTCGGGTGTGTTAATAGCCTCCTGTCACATGTGCCGGATTAGACTGAGGAACGCAGCTTGCGCAGGCGGGGTCGCCCACGTACAAGCTTAGATGTGACATAATGCTTTAAAC
GACCCGTCTGAAACACGGACAGAGTCTACATATCTGCGAGTGTTTGGGTGTCAAACCCAGCGCGAATGAAAGTGAACGTAGAGGATCCGCAAGGGAGCACCTTCGACCGATCCGGAAGAATCT
>ITC_N
CTATGGTGACTGCGGAGATCATTTCTGTTTCTATAGCTTTGCGTGCGCTGCCTTTACGGGCCTGCGCTGCAAGCGAAAACCTTATCACACTTGTGTTTTTATTGAAGACAACTTTGCTTTGGTCGAATGGCGTAAAAACC
TTTGGCCAGAGGATATTAAACTCAAACTAAATTTTTATTTAACAATATGTCAGAATTTTAATCTATTAATTTAGATAACTAATATTCAAAACTTTCAACAACGGATCTCTTGGTTCTCGCATCGATGAAGAACGCAGCGAAAT
GCGATAAGTATTGTGAATTGCAGATTTTCGTGAATCATCGAATCTTTGAACGCACATTGCGCCCTCTAGTATTCTAGAGGGCATGCCTGTTTGAGCGTCATTTCTCTCTCATAACCCTTCTGGGTTTTGGTTGTGAACGG
TAAACCTTTTGGTTTTCGTTTGAAATGAATTGACCGGCTGGCTTTCAATGGCTTGTTATGTTTATTCAATGTATTAGGTTCTTCCAACTCGTTGATTGACAAAGCAAATACCACGAAAGATTCGGCTTGTCGAACTTTACCT
TAAAGTTTGACCTCAAATCAGGTAGGATTACCCGCTGAACTTAAGCATATCAATAAGCGGAGGAAAAGAAACCAACAGGGATTGCCTTAGTAGCGGCGAGTGAAGCGGCAAGAGCTCAAATTTGAAATCTGGTACCTT
CGGTGCCCGAGTTGTAATTTGAAGAAGGTATTCTTTAGTTTGGCCCCCGTCTATGTTCCTTGGAACAGGACGTCATAGAGGGTGAGAATCCCGTGCGATGGGGTGTCCATTCTTTGTAAGATTCCTTCGACGAGTCGA
GTTGTTTGGGAATGCAGCTCTAGTGGGTGGTAAATTCCATCTAAAGCTAAATATTGGCGAGAGACCGATAGCGAACAAGTACAGTGATGGAAAGATGAAAAGAACTTTGAAAAGAGAGTGAAAAAGTACGTGAAATTGT
TGAAAGGAAGGGTATTTGATCAGACTTGGTATTAGAATATGGTTTCTCCTTGTGGGTTTCCCTCTATTCTTTTACTGGGCCAGCATCGGTTCGGATGGTAAGATATTGCGATTGATGTGGCTCTTCGGAGTGTATAGCTT
TCGTCGATATGCCTGTCTGACGAGGACTGCGTCTTTTGACTAGATGCTGCGTATGATAAATACCGCCGTCTGAACATGACAGGAGTCTACGTCTATGGCGAGTGTTAGGTGTAAACCTTACCGCGTTATTGGAAGTTGA
ACGTAGGTAG
>ITC_O
TTACAGTATTCTATTGCCAGCGCTTAATTGCGCGGCGATAAACCTTACACACTTTGTTTTTATTATTAAAGACACCATTGCTTTGGTGGATGAGTGTAAAAGCTTGTCCGCCAGAGGTTAACTAAAACTCAATTATTTAAA
GGTAACTTTTTAAATTGTCAATAATAATTTGTCTGATTAATTTCGGAGAAAACAAATATTTAAAACTTTCAACAACGGATCTCTTGGTTCTCGCATCGATGAAGAACGCAGCGAATTGCGATAAGTATTGTGAATTGCAGAA
TTTCGTGAATCATCGAATCTTTGAACGCACATTGCACCCTGTGGTATTCCACAGGGTATGCCTGTTTGAGCGTCATTTCTCTCTCAAAACATTAGTTTTGGTATTGAGTGATACTCGGGTTTTAGGCTTGAGTTTGCTTGA
AAAGTATTGGCAAGGGAAGTATTTCTTGTGAAAGCTATTTATTCAATGTATTAGGTTTATCCAACTCGTTGATTGATAGACGGAACTCGTTTTGCTATTAGGCCCGGCCTTACAACAAACAAACCAAAGTTTGACCTCAAA
TCAGGTAGGATTACCCGCTGAACTTAAGCATATCAATAAGCGGAGGAAAAGAAACCAACAGGGATTACCTTAGTAACGGCGAGTGAAGCGGTAAAAGCTCAAATTTGAAATCTAGCACTTTCAGTGTTCGAGTTGTAAT
TTGAAGAAGGTATCTTTGGAGTTGGCCCCCGTCTATGTTCCTTGGAACAGGACGTCACAGAGGGTGAGAATCCCGTGCGATGGGGAGACCAATTCTATGTAAAGTTCCTTCGAAGAGTCGAGTTGTTTGGGAATGCAG
CTCTAAGTGGGTGGTAAATTCCATCTAAAGCTAAATATTGGCGAGAGACCGATAGCGAACAAGTACAGTGATGGAAAGATGAAAAGAACTTTGAAAAGAGAGTGAAAAAGTACGTGAAATTGTTG
>ITC_S
CTAGGGGGACTGCGGAGATCATTCATGAATTTGGACCTCTGTGTCCTAAATCTCATCCTTCAATACCTCTGTGAACTGTTGGCCCTCGGGCCTATTACAAACATCAGTGTAATGAACGTCATACATATATAACAATTATA
AAACTTTCAACAACGGATCTCTTGGCTCTCGCATCGATGAAGAACGCAGCGAAATGCGATAAGTAATGTGAATTGCAGAATTCAGTGAATCATCGAATCTTTGAACGCACCTTGCACCTTTTGGTACTCCGAAAGGTAT
GCCTGTTTGAGTGTCATGAAATCTCAATCCCCTCTGGTTTCATTACCAGGGGTGGACTTGGACTTGGGCGTCTGCCATATTGCTTGGCTCGCCTCAAATGTGTCAGTGGGATCTAGCATCCACGGCCCGACGTAATAA
GTTTCGTCTGGCCCGGTGTCTGAGCCTGCTCATAACCAACCATATCTTCTGACTCTGACCTCAAATCAGGTAGGGCTACCCGCTGAACTTAAGCATATCAATAAGCGGAGGAAAAGAAACTAACAAGGATTCCCCTAGT
AACGGCGAGTGAACAGGGAAGAGCTCAAATTTGAAATCTGGCGTCCTCAGGGCGTCCGAGTTGTAATCTATAGAGTCGTTTTCCGTGCCGGACCGTATCCAAGTTCCTTGGAATAGGATATCAAAGAGGGTGACAATC
CCGTGCTTGATACGACGACCGGTGCTCTGTGATACGTCTTCTACGAGTCGAGTTGTTTGGGAATGCAGCTCAAAATGGGTGGTGAACTCCATCTAAAGCTAAATATTGGCGAGAGACCGATAGCGAACAAGTACCGTG
AGGGAAAGATGAAAAGCACTTTGGAAAGAGAGTTAAACAGTACGTGAAATTGTTGAAAGGGAAACGATTGAAGTCAGTCGTGTCCGAGAGGCTCAGCCGGTTCTGCCGGTGTATTCCTCTCGGACGGGTCAACATCA
GTTTTGACCGGTGGATAAGGCGATGGGAAGGTGGCACCCTCGGGTGTGTTATAGCCCGTCGTCGCATACATCGGTCGAGACTGAGGAATGCAGCTCGCCTTATGGGCCGGGGTTCGCCCACGTTCGAGCTTAGGAT
GTTGACATATGGCTTTAAACGACCCGTCTGAAACACGGACCAGGAGTCTAACATATCTGCGAGTGTTGGGTGTCAAACCAGCGCGTATGAGGTAACGTAAGGAGGGATCGTAGGAGCACTTTCGACGATCTGGATCT
CTCTGTGTGAATGGGATTTGGAG
