# Supplementary material for: Distinct Taphrina strains from the phyllosphere of birch exhibiting a range of witches' broom disease symptoms
Source: Environ Microbiol. 2022 May 17;24(8):3549–64. doi: 10.1111/1462-2920.16037 (PMC9545635; doi:10.1111/1462-2920.16037)
Supplement: Supplementary file 7 — Table S2. Full list of all strains isolated. Abbreviations used: nd, no data; np, no ITS PCR product. [file EMI-24-3549-s008.pdf]

Supplemental Table S2: Full list of all strains isolated. [a] Samples are defined in Table 1. [B]Strain growth was measured by drop inoculations of serial dilutions on 0.2 X PDA plates and incubation at the indicated temperature for four days then were visually assessed and characterized as noted: +++, strong growth; ++, intermediate growth; + weak growth. [c]Nuclear rRNA internal transcribed spacer (ITS) PCR products were used as cleaved amplified polymorphic sequence (CAPS) marker, called ITS Taq I CAPS (ITC), by digestion with the restriction endonuclease Taq I. ITC types represent distinct banding patters (ITC-A to ITC-S; see Table S1) Abbreviations used: ui, unidentified, np, no PCR product. [d]RGR (Rco1 Gyg7 Rsa1) is a CAPS marker that amplifies the polymorphic intergenic region between the *Taphrina betulina* Gyg7 and Rco1 genes and is digested with the restriction nuclease RsaI. It is used to differential between *T. betulina* strains and identifies four different banding patters RGR-0, no PCR product, and RGR-1 to RGR-3, which represent banding pattern variants I-III, respectively. See Supplemental Figure 3 for further details on the design of CAPS markers. Abbreviations used: nt, not tested; nd, no data; np, no ITS PCR product; ui, unidentified.

Strain	Location	Tree phenotype	Isolation source	Sample[a]	Growth 30°C[b]	Growth 21°C[b]	ITC type[c]	RGR type[d]
**1**	Pihlajisto	Small healthy	Healthy appearing leaf	A	nd	nd	np	nt
**2**	Pihlajisto	Small healthy	Healthy appearing leaf	A	-	+++	ITC-E	nt
**3**	Pihlajisto	Small healthy	Healthy appearing leaf	A	-	+++	ITC-E	nt
**4**	Pihlajisto	Small healthy	Healthy appearing leaf	A	-	+++	ITC-E	nt
**5**	Pihlajisto	Small healthy	Healthy appearing leaf	A	-	+++	ITC-E	nt
**6**	Pihlajisto	Small healthy	Healthy appearing leaf	A	-	+++	ITC-E	nt
**7**	Pihlajisto	Small healthy	Healthy appearing leaf	A	-	+++	ITC-F	nt
**8**	Pihlajisto	Small healthy	Healthy appearing leaf	A	-	+++	ITC-E	nt
**9**	Pihlajisto	Small healthy	Healthy appearing leaf	A	-	+	ITC-E	nt

**10**	Pihlajisto	Small healthy	Healthy appearing leaf	A	-	+	ITC-E	nt
**11**	Pihlajisto	Small healthy	Healthy appearing leaf	A	-	+	ITC-C	RGR-1
**12**	Pihlajisto	Small healthy	Healthy appearing leaf	A	-	+++	ITC-E	nt
**13**	Pihlajisto	Small healthy	Healthy appearing leaf	A	-	+	ITC-E	nt
**14**	Pihlajisto	Small healthy	Healthy appearing leaf	A	-	+++	ITC-E	nt
**15**	Pihlajisto	Small healthy	Healthy appearing leaf	A	-	+++	ITC-E	nt
**16**	Pihlajisto	Small healthy	Healthy appearing leaf	A	-	+++	ITC-E	nt
**17**	Pihlajisto	Small healthy	Healthy appearing leaf	A	-	+++	ITC-E	nt
**18**	Pihlajisto	Small healthy	Healthy appearing leaf	A	-	+	ITC-E	nt
**19**	Pihlajisto	Small healthy	Healthy appearing leaf	A	-	+++	ITC-D	RGR-0
**20**	Pihlajisto	Small healthy	Healthy appearing leaf	A	-	+++	ITC-D	RGR-2
**21**	Pihlajisto	Small healthy	Healthy appearing leaf	A	+	+++	ITC-D	RGR-2
**22**	Pihlajisto	Small healthy	Healthy appearing leaf	A	-	+++	ITC-D	RGR-2
**23**	Pihlajisto	Small healthy	Healthy appearing leaf	A	-	+++	ITC-F	nt
**24**	Pihlajisto	Small healthy	Healthy appearing leaf	A	-	+++	ITC-E	nt
**25**	Viikinkaari	Large birch with elongated brooms	Broom leaf small and deformed	B	-	+++	ITC-D	RGR-2

**26**	Viikinkaari	Large birch with elongated brooms	Broom leaf small and deformed	B	-	+++	ITC-D	RGR-2
**27**	Viikinkaari	Large birch with elongated brooms	Broom leaf small and deformed	B	+	+	ITC-D	RGR-0
**28**	Viikinkaari	Large birch with elongated brooms	Broom leaf small and deformed	B	+	+++	np	nt
**29**	Viikinkaari	Large birch with elongated brooms	Broom leaf small and deformed	B	-	+++	ITC-H	nt
**30**	Viikinkaari	Large birch with elongated brooms	Broom leaf small and deformed	B	-	+++	ITC-D	RGR-2
**31**	Viikinkaari	Large birch with elongated brooms	Broom leaf small and deformed	B	-	+++	ITC-D	RGR-0
**32**	Viikinkaari	Large birch with elongated brooms	Broom leaf small and deformed	B	-	++	ITC-D	RGR-2
**33**	Viikinkaari	Large birch with elongated brooms	Broom leaf small and deformed	B	-	+++	ITC-D	RGR-2
**34**	Viikinkaari	Large birch with elongated brooms	Broom leaf small and deformed	B	-	+	ITC-D	RGR-2
**35**	Viikinkaari	Large birch with elongated brooms	Broom leaf small and deformed	B	+	+++	ITC-F	nt
**36**	Viikinkaari	Large birch with elongated brooms	Broom leaf small and deformed	B	-	+++	ITC-D	RGR-2

**37**	Viikinkaari	Large birch with elongated brooms	Broom leaf small and deformed	B	-	+++	ITC-D	RGR-2
**38**	Viikinkaari	Large birch with elongated brooms	Broom leaf small and deformed	B	-	+++	ui	nt
**39**	Viikinkaari	Large birch with elongated brooms	Broom leaf small and deformed	B	-	-	ITC-D	RGR-0
**40**	Viikinkaari	Large birch with elongated brooms	Broom leaf small and deformed	B	-	+++	ITC-N	nt
**41**	Viikinkaari	Large birch with elongated brooms	Broom leaf small and deformed	B	+	+++	ITC-O	nt
**42**	Viikinkaari	Large birch with elongated brooms	Broom leaf small and deformed	B	+	+++	np	nt
**43**	Viikinkaari	Large birch with elongated brooms	Broom leaf small and deformed	B	-	-	ITC-D	RGR-0
**44**	Viikinkaari	Large birch with elongated brooms	Broom leaf small and deformed	B	-	+++	ITC-D	RGR-2
**45**	Viikinkaari	Large birch with elongated brooms	Broom leaf small and deformed	B	+	+++	np	nt
**46**	Viikinkaari	Large birch with elongated brooms	Broom leaf small and deformed	B	-	-	ui	nt
**47**	Viikinkaari	Large birch with elongated brooms	Broom leaf small and deformed	B	-	-	ui	nt

**48**	Viikinkaari	Large birch with elongated brooms	Broom leaf small and deformed	B	-	-	ui	nt
**49**	Viikinkaari	Large birch with elongated brooms	Broom leaf small and deformed	B	-	-	ITC-D	RGR-2
**50**	Viikinkaari	Large birch with elongated brooms	Broom leaf small and deformed	B	-	+++	ITC-D	RGR-2
**51**	Viikinkaari	Large birch with elongated brooms	Broom leaf small and deformed	B	-	-	ITC-D	RGR-0
**52**	Viikinkaari	Large birch with elongated brooms	Broom leaf small and deformed	B	+	+++	np	nt
**53**	Viikinkaari	Large birch with elongated brooms	Broom leaf small and deformed	B	-	++	ITC-D	RGR-2
**54**	Viikinkaari	Large birch with elongated brooms	Broom tree leaf with gray small spot	C	+	+++	ITC-F	nt
**55**	Viikinkaari	Large birch with elongated brooms	Broom tree leaf with gray small spot	C	+	+	ITC-F	nt
**56**	Viikinkaari	Large birch with elongated brooms	Broom tree leaf with gray small spot	C	+	+++	ui	nt
**57**	Viikinkaari	Large birch with elongated brooms	Broom tree leaf with gray small spot	C	-	+++	ITC-D	RGR-2
**58**	Viikinkaari	Large birch with elongated brooms	Broom tree leaf with gray small spot	C	-	+++	ITC-D	RGR-3

**59**	Viikinkaari	Large birch with elongated brooms	Broom tree leaf with gray small spot	C	-	+++	ITC-D	RGR-2
**60**	Viikinkaari	Large birch with elongated brooms	Broom tree leaf with gray small spot	C	-	+++	ITC-I	nt
**61**	Viikinkaari	Large birch with elongated brooms	Broom tree leaf with gray small spot	C	-	++	ui	nt
**62**	Viikinkaari	Large birch with elongated brooms	Broom tree leaf with gray small spot	C	-	+++	ITC-D	RGR-2
**63**	Viikinkaari	Large birch with elongated brooms	Broom tree leaf with gray small spot	C	-	+	ITC-C	RGR-2
**64**	Viikinkaari	Large birch with elongated brooms	Broom tree leaf with gray small spot	C	-	+	ITC-D	RGR-0
**65**	Viikinkaari	Large birch with elongated brooms	Broom tree leaf with gray small spot	C	+	+++	ui	nt
**66**	Viikinkaari	Large birch with elongated brooms	Broom tree leaf with gray small spot	C	+	+++	ui	nt
**67**	Viikinkaari	Large birch with elongated brooms	Broom tree leaf with gray small spot	C	+	+++	ITC-D	RGR-2
**68**	Viikinkaari	Large birch with elongated brooms	Broom tree leaf with gray small spot	C	+	+++	ITC-C	RGR-2
**69**	Viikinkaari	Large birch with elongated brooms	Broom tree leaf with gray small spot	C	+	+++	ITC-D	RGR-2

**70**	Viikinkaari	Large birch with elongated brooms	Broom tree leaf with gray small spot	C	+	+++	np	nt
**71**	Viikinkaari	Large birch with elongated brooms	Broom tree leaf with gray small spot	C	+	+++	np	nt
**72**	Viikinkaari	Large birch with elongated brooms	Broom tree leaf with gray small spot	C	-	+++	ITC-D	RGR-2
**73**	Viikinkaari	Large birch with elongated brooms	Broom tree leaf with gray small spot	C	+	+++	ITC-F	nt
**74**	Viikinkaari	Large birch with elongated brooms	Broom tree leaf with gray small spot	C	-	+++	ITC-D	RGR-2
**75**	Viikinkaari	Large birch with elongated brooms	Broom tree leaf with gray small spot	C	-	+++	ITC-D	RGR-2
**76**	Viikinkaari	Large birch with elongated brooms	Broom tree leaf with gray small spot	C	+	+++	ui	nt
**77**	Pihlajisto	Small healthy	Healthy appearing leaf	A	-	+++	np	nt
**78**	Pihlajisto	Small healthy	Healthy appearing leaf	A	+	+++	ITC-I	nt
**79**	Pihlajisto	Small healthy	Healthy appearing leaf	A	+	+++	ITC-I	nt
**80**	Pihlajisto	Small healthy	Healthy appearing leaf	A	+	+++	ITC-E	nt
**81**	Pihlajisto	Small healthy	Healthy appearing leaf	A	+	+++	ITC-E	nt
**82**	Viikinkaari	Large birch with elongated brooms	Broom tree leaf with gray small spot	C	-	+++	ITC-D	RGR-1

**83**	Viikinkaari	Large birch with elongated brooms	Broom tree leaf with gray small spot	C	-	+++	ITC-D	RGR-0
**84**	Viikinkaari	Large birch with elongated brooms	Broom leaf small and deformed	B	+	+++	ui	nt
**85**	Viikinkaari	Large birch with elongated brooms	Broom leaf small and deformed	B	-	+++	ITC-C	RGR-0
**86**	Vartioharju (VTH)	Large birch hanging phenotype elongated brooms, heavily diseased (VTHS1)	Broom tree leaf with gray small spots and yellow spot and deficient growth (BTL)	D	-	+++	ITC-A	nt
**87**	Vartioharju (VTH)	Large birch hanging phenotyp) with elongated brooms, heavily diseased (VTHS1)	Broom tree leaf with gray small spots and yellow spot and deficient growth (BTL)	D	+	+++	ITC-F	nt
**88**	Vartioharju (VTH)	Large birch hanging phenotype elongated brooms, heavily diseased (VTHS1)	Broom tree leaf with gray small spots and yellow spot and deficient growth (BTL)	D	+	+++	ITC-F	nt
**89**	Vartioharju (VTH)	Large birchhanging phenotype elongated	Broom tree leaf with gray small spots and yellow spot and	D	+	+++	ITC-F	nt

		brooms, heavily diseased (VTHS1)	deficient growth (BTL)					
**90**	Vartioharju (VTH)	Large birch hanging phenotype with elongated brooms, heavily diseased (VTHS1)	Broom tree leaf with gray small spots and yellow spot and deficient growth (BTL)	D	+	+++	ITC-F	nt
**91**	Vartioharju (VTH)	Large birch hanging phenotype elongated brooms, heavily diseased (VTHS1)	Broom tree leaf with gray small spots and yellow spot and deficient growth (BTL)	D	+	+++	ITC-F	nt
**92**	Vartioharju (VTH)	Large birch hanging phenotype with elongated brooms, heavily diseased (VTHS1)	Broom tree leaf with gray small spots and yellow spot and deficient growth (BTL)	D	+	+++	ITC-F	nt
**93**	Vartioharju (VTH)	Large birch(hanging phenotype elongated brooms, heavily diseased (VTHS1)	Broom tree leaf with gray small spots and yellow spot and deficient growth (BTL)	D	+	+++	ITC-A	nt
**94**	Vartioharju (VTH)	Large birch hanging phenotype	Broom tree leaf with gray small spots and yellow spot and	D	+	+++	ITC-A	nt

		elongated brooms, heavily diseased (VTHS1)	deficient growth (BTL)					
**95**	Vartioharju (VTH)	Large birch hanging phenotype elongated brooms, heavily diseased (VTHS1)	Broom tree leaf with gray small spots and yellow spot and deficient growth (BTL)	D	+	+++	ITC-F	nt
**96**	Vartioharju (VTH)	Large birch hanging phenotype elongated brooms, heavily diseased (VTHS1)	Broom tree leaf with gray small spots and yellow spot and deficient growth (BTL)	D	+	+++	ITC-F	nt
**97**	Vartioharju (VTH)	Large birch hanging phenotype elongated brooms, heavily diseased (VTHS1)	Broom tree leaf with gray small spots and yellow spot and deficient growth (BTL)	D	-	+++	ITC-F	nt
**98**	Vartioharju (VTH)	Large birch(hanging phenotype) with elongated brooms, heavily diseased (VTHS1)	Broom tree leaf with gray small spots and yellow spot and deficient growth (BTL)	D	-	+	ITC-F	nt
**99**	Vartioharju (VTH)	Large birch hanging	Broom tree leaf with gray small spots	D	+++	+++	np	nt

		phenotype elongated brooms, heavily diseased (VTHS1)	and yellow spot and deficient growth (BTL)					
**100**	Vartioharju (VTH)	Large birch hanging phenotype elongated brooms, heavily diseased (VTHS1)	Broom tree leaf with gray small spots and yellow spot and deficient growth (BTL)	D	+++	+++	np	nt
**101**	Vartioharju (VTH)	Large birch hanging phenotype elongated brooms, heavily diseased (VTHS1)	Broom tree leaf with gray small spots and yellow spot and deficient growth (BTL)	D	+++	+++	np	nt
**102**	Vartioharju (VTH)	Large birch hanging phenotype elongated brooms, heavily diseased (VTHS1)	Broom tree leaf with gray small spots and yellow spot and deficient growth (BTL)	D	+++	+++	np	nt
**103**	Vartioharju (VTH)	Large birch hanging phenotype elongated brooms, heavily diseased (VTHS1)	Broom tree leaf with gray small spots and yellow spot and deficient growth (BTL)	D	+++	+++	np	nt

**104**	Vartioharju (VTH)	Large birch hanging phenotype elongated brooms, heavily diseased (VTHS1)	Broom leaf gray spots and yellow spot and deficient growth	E	+++	+++	np	nt
**105**	Vartioharju (VTH)	Large birch hanging phenotype elongated brooms, heavily diseased (VTHS1)	Broom leaf gray spots and yellow spot and deficient growth	E	+++	+++	np	nt
**106**	Vartioharju (VTH)	Large birch hanging phenotype elongated brooms, heavily diseased (VTHS1)	Broom leaf gray spots and yellow spot and deficient growth	E	-	+++	ITC-L	nt
**107**	Vartioharju (VTH)	Large birch hanging phenotype elongated brooms, heavily diseased (VTHS1)	Broom leaf gray spots and yellow spot and deficient growth	E	++	++	np	nt
**108**	Vartioharju (VTH)	Large birch hanging phenotype elongated brooms, heavily diseased	Broom leaf gray spots and yellow spot and deficient growth	E	++	+	np	nt

		(VTHS1)						
**109**	Vartioharju (VTH)	Large birch hanging phenotype with elongated brooms, heavily diseased (VTHS1)	Broom leaf gray spots and yellow spot and deficient growth	E	-	+++	ITC-L	nt
**110**	Vartioharju (VTH)	Large birch hanging phenotype elongated brooms, heavily diseased (VTHS1)	Broom leaf gray spots and yellow spot and deficient growth	E	++	+++	np	nt
**111**	Vartioharju (VTH)	Large birch hanging phenotype elongated brooms, heavily diseased (VTHS1)	Broom leaf gray spots and yellow spot and deficient growth	E	-	+++	np	nt
**112**	Vartioharju (VTH)	Large birch hanging phenotype elongated brooms, heavily diseased (VTHS1)	Broom leaf gray spots and yellow spot and deficient growth	E	+	+++	ITC-D	RGR-0
**113**	Vartioharju (VTH)	Large birch hanging phenotype elongated brooms, heavily	Broom leaf gray spots and yellow spot and deficient growth	E	++	+++	np	nt

		diseased (VTHS1)						
**114**	Vartioharju (VTH)	Large birch hanging phenotype elongated brooms, heavily diseased (VTHS1)	Broom leaf gray spots and yellow spot and deficient growth	E	++	+++	np	nt
**115**	Vartioharju (VTH)	Large birch hanging phenotype elongated brooms, heavily diseased (VTHS1)	Broom leaf gray spots and yellow spot and deficient growth	E	++	+++	np	nt
**116**	Vartioharju (VTH)	Large birch hanging phenotype elongated brooms, heavily diseased (VTHS1)	Broom leaf gray spots and yellow spot and deficient growth	E	++	+++	np	nt
**117**	Vartioharju (VTH)	Large birch hanging phenotype elongated brooms, heavily diseased (VTHS1)	Broom leaf gray spots and yellow spot and deficient growth	E	++	+++	ITC-M	nt
**118**	Vartioharju (VTH)	Large birch hanging phenotype elongated	Broom leaf gray spots and yellow spot and deficient growth	E	-	+	ITC-M	nt

		brooms, heavily diseased (VTHS1)						
**119**	Vartioharju (VTH)	Large birch hanging phenotype elongated brooms, heavily diseased (VTHS1)	Broom leaf gray spots and yellow spot and deficient growth	E	++	+++	np	nt
**120**	Vartioharju (VTH)	Large birch hanging phenotype elongated brooms, heavily diseased (VTHS1)	Broom leaf gray spots and yellow spot and deficient growth	E	++	+++	ITC-D	RGR-0
**121**	Vartioharju (VTH)	Large birch hanging phenotype elongated brooms, heavily diseased (VTHS1)	Broom leaf gray spots and yellow spot and deficient growth	E	-	+++	ITC-F	nt
**122**	Vartioharju (VTH)	Large birch hanging phenotype elongated brooms, heavily diseased (VTHS1)	Broom leaf gray spots and yellow spot and deficient growth	E	-	-	np	nt
**123**	Vartioharju (VTH)	Large birch hanging phenotype	Broom leaf gray spots and yellow	E	++	+++	np	nt

		elongated brooms, heavily diseased (VTHS1)	spot and deficient growth					
**124**	Vartioharju (VTH)	Large birch hanging phenotype elongated brooms, heavily diseased (VTHS1)	Broom leaf gray spots and yellow spot and deficient growth	E	++	+++	np	nt
**125**	Vartioharju (VTH)	Large birch hanging phenotype elongated brooms, heavily diseased (VTHS1)	Broom leaf gray spots and yellow spot and deficient growth	E	++	+++	ITC-M	nt
**126**	Vartioharju (VTH)	Large birch hanging phenotype elongated brooms, heavily diseased (VTHS1)	Broom leaf gray spots and yellow spot and deficient growth	E	++	+++	ITC-M	nt
**127**	Vartiokylä (VTKR2)	Large healthy birch (near heavily diseased tree)	Healthy appearing leaf	F	-	++	ITC-A	nt
**128**	Vartiokylä (VTKR2)	Large healthy birch (near heavily diseased tree)	Healthy appearing leaf	F	-	+	ITC-E	nt

**129**	Vartiokylä (VTKR2)	Large healthy birch (near heavily diseased tree)	Healthy appearing leaf	F	-	+++	ITC-D	RGR-2
**130**	Vartiokylä (VTKR2)	Large healthy birch (near heavily diseased tree)	Healthy appearing leaf	F	-	+	ITC-E	nt
**131**	Vartiokylä (VTKR2)	Large healthy birch (near heavily diseased tree)	Healthy appearing leaf	F	-	++	ITC-A	nt
**132**	Vartiokylä (VTKR2)	Large healthy birch (near heavily diseased tree)	Healthy appearing leaf	F	-	++	ITC-F	nt
**133**	Vartiokylä (VTKR2)	Large healthy birch (near heavily diseased tree)	Healthy appearing leaf	F	-	+++	ITC-A	nt
**134**	Vartiokylä (VTKR2)	Large healthy birch (near heavily diseased tree)	Healthy appearing leaf	F	-	+	ITC-A	nt
**135**	Vartiokylä (VTKR2)	Large healthy birch (near heavily diseased tree)	Healthy appearing leaf	F	-	+++	ITC-A	nt
**136**	Vartiokylä (VTKR2)	Large healthy birch (near heavily diseased tree)	Healthy appearing leaf	F	-	+++	ITC-F	nt
**137**	Vartiokylä (VTKR2)	Large healthy birch (near	Healthy appearing leaf	F	-	+++	ITC-A	nt

		heavily diseased tree)						
**138**	Vartiokylä (VTKR2)	Large healthy birch (near heavily diseased tree)	Healthy appearing leaf	F	-	+++	ITC-A	nt
**139**	Vartiokylä (VTKR2)	Large healthy birch (near heavily diseased tree)	Healthy appearing leaf	F	-	+++	ITC-A	nt
**140**	Vartiokylä (VTKR2)	Large healthy birch (near heavily diseased tree)	Healthy appearing leaf	F	-	+++	ITC-A	nt
**141**	Vartiokylä (VTKR2)	Large healthy birch (near heavily diseased tree)	Healthy appearing leaf	F	-	+++	ITC-A	nt
**142**	Vartiokylä (VTKR2)	Large healthy birch (near heavily diseased tree)	Healthy appearing leaf	F	-	+++	ITC-A	nt
**143**	Vartiokylä (VTKR2)	Large healthy birch (near heavily diseased tree)	Healthy appearing leaf	F	-	+++	ITC-A	nt
**144**	Vartiokylä (VTKR2)	Large healthy birch (near heavily diseased tree)	Healthy appearing leaf	F	-	+++	ITC-A	nt
**145**	Vartiokylä (VTKR2)	Large healthy birch (near heavily diseased tree)	Healthy appearing leaf	F	+	+++	np	nt

**146**	Vartiokylä (VTKR2)	Large healthy birch (near heavily diseased tree)	Healthy appearing leaf	F	-	+++	ITC-I	nt
**147**	Vartiokylä	Large birch broom like tumors no shoot elongation from tumors (VTKS1).	Broom tree leaf with chlorotic regions and gray spots	G	-	+++	ITC-F	nt
**148**	Vartiokylä	Large birch broom like tumors no shoot elongation from tumors (VTKS1).	Broom tree leaf with chlorotic regions and gray spots	G	-	+++	ITC-A	nt
**149**	Vartiokylä	Large birch broom like tumors no shoot elongation from tumors (VTKS1).	Broom tree leaf with chlorotic regions and gray spots	G	-	+++	ITC-A	nt
**150**	Vartiokylä	Large birch broom like tumors no shoot elongation from tumors (VTKS1).	Broom tree leaf with chlorotic regions and gray spots	G	-	+++	ITC-F	nt
**151**	Vartiokylä	Large birch broom like tumors no shoot elongation from tumors (VTKS1).	Broom tree leaf with chlorotic regions and gray spots	G	-	+++	ITC-D	RGR-2

**152**	Vartiokylä	Large birch broom like tumors no shoot elongation from tumors (VTKS1).	Broom tree leaf with chlorotic regions and gray spots	G	++	+++	ITC-F	nt
**153**	Vartiokylä	Large birch broom like tumors no shoot elongation from tumors (VTKS1).	Broom tree leaf with chlorotic regions and gray spots	G	-	+++	ITC-F	nt
**154**	Vartiokylä	Large birch broom like tumors no shoot elongation from tumors (VTKS1).	Broom tree leaf with chlorotic regions and gray spots	G	-	+++	ITC-F	nt
**155**	Vartiokylä	Large birch broom like tumors no shoot elongation from tumors (VTKS1).	Broom tree leaf with chlorotic regions and gray spots	G	-	+++	ITC-F	nt
**156**	Vartiokylä	Large birch broom like tumors no shoot elongation from tumors (VTKS1).	Broom tree leaf with chlorotic regions and gray spots	G	-	+++	ITC-F	nt
**157**	Vartiokylä	Large birch broom like tumors no shoot elongation from	Broom tree leaf with chlorotic regions and gray spots	G	-	+++	ITC-F	nt

		tumors (VTKS1).						
**158**	Vartiokylä	Large birch broom like tumors no shoot elongation from tumors (VTKS1).	Broom tree leaf with chlorotic regions and gray spots	G	-	+++	ITC-F	nt
**159**	Vartiokylä	Large birch broom like tumors no shoot elongation from tumors (VTKS1).	Broom tree leaf with chlorotic regions and gray spots	G	+	+	ITC-E	nt
**160**	Vartiokylä	Large birch broom like tumors no shoot elongation from tumors (VTKS1).	Broom tree leaf with chlorotic regions and gray spots	G	-	+++	ITC-A	nt
**161**	Vartiokylä	Large birch broom like tumors no shoot elongation from tumors (VTKS1).	Broom tree leaf with chlorotic regions and gray spots	G	+++	+++	np	nt
**162**	Vartiokylä	Large birch broom like tumors no shoot elongation from tumors (VTKS1).	Broom tree leaf with chlorotic regions and gray spots	G	-	+++	ITC-A	nt

**163**	Vartiokylä	Large birch broom like tumors no shoot elongation from tumors (VTKS1).	Broom tree leaf with chlorotic regions and gray spots	G	-	+++	ITC-E	nt
**164**	Vartiokylä	Large birch broom like tumors no shoot elongation from tumors (VTKS1).	Broom tree leaf with chlorotic regions and gray spots	G	+	+++	ITC-E	nt
**165**	Vartiokylä	Large birch broom like tumors no shoot elongation from tumors (VTKS1).	Broom tree leaf with chlorotic regions and gray spots	G	-	+++	ITC-E	nt
**166**	Vartiokylä	Large birch broom like tumors no shoot elongation from tumors (VTKS1).	Broom tree leaf with chlorotic regions and gray spots	G	-	-	np	nt
**167**	Vartiokylä	Large birch broom like tumors no shoot elongation from tumors (VTKS1).	Broom tree leaf with chlorotic regions and gray spots	G	+++	+++	np	nt
**168**	Herttoniemi	HERS1 Large planted birch lots of elongated small brooms	Broom tree leaf with gray spots	H	-	+++	ITC-A	nt

**169**	Herttoniemi	HERS1 Large planted birch lots of elongated small brooms	Broom tree leaf with gray spots	H	-	+++	ITC-A	nt
**170**	Herttoniemi	HERS1 Large planted birch lots of elongated small brooms	Broom tree leaf with gray spots	H	-	+++	ITC-A	nt
**171**	Herttoniemi	HERS1 Large planted birch lots of elongated small brooms	Broom tree leaf with gray spots	H	-	++	ITC-E	nt
**172**	Herttoniemi	HERS1 Large planted birch lots of elongated small brooms near to gate	Broom tree leaf with gray spots	H	-	++	ITC-F	nt
**173**	Herttoniemi	HERS1 Large planted birch lots of elongated small brooms	Broom tree leaf with gray spots	H	-	+++	ITC-A	nt
**174**	Herttoniemi	HERS1 Large planted birch lots of elongated small brooms	Broom tree leaf with gray spots	H	+	+++	ITC-A	nt
**175**	Herttoniemi	HERS1 Large planted birch lots of elongated small brooms	Broom tree leaf with gray spots	H	-	+++	ITC-A	nt
**176**	Herttoniemi	HERS1 Large planted birch lots of elongated small brooms	Broom tree leaf with gray spots	H	-	+++	ITC-A	nt

**177**	Herttoniemi	HERS1 Large planted birch lots of elongated small brooms	Broom tree leaf with gray spots	H	-	+++	ITC-A	nt
**178**	Herttoniemi	HERS1 Large planted birch lots of elongated small brooms	Broom tree leaf with gray spots	H	+	+++	ITC-A	nt
**179**	Herttoniemi	HERS1 Large planted birch, many elongated small brooms	Broom tree leaf with gray spots	H	-	+++	ITC-A	nt
**180**	Herttoniemi	HERS1 Large planted birch many elongated small brooms	Broom tree leaf with gray spots	H	-	+++	ITC-F	nt
**181**	Herttoniemi	HERS1 Large planted birch lots of elongated small brooms	Broom tree leaf with gray spots	H	-	+++	ITC-A	nt
**182**	Herttoniemi	HERS1 Large planted birch lots of elongated small brooms	Broom tree leaf with gray spots	H	-	+++	ITC-A	nt
**183**	Herttoniemi	HERS1 Large planted birch lots of elongated small brooms	Broom tree leaf with gray spots	H	-	+++	ITC-F	nt
**184**	Herttoniemi	HERS1 Large planted birch lots of elongated small brooms	Broom tree leaf with gray spots	H	-	+++	ITC-F	nt
**185**	Herttoniemi	HERS1 Large planted birch	Broom tree leaf with gray spots	H	-	++	ITC-F	nt

		lots of elongated small brooms						
**186**	Herttoniemi	HERS1 Large planted birch lots of elongated small brooms	Broom tree leaf with gray spots	H	+	+++	ui	nt
**187**	Herttoniemi	HERS1 Large planted birch lots of elongated small brooms	Broom tree leaf with gray spots	H	-	-	np	nt
**188**	Herttoniemi	HERS1 Large planted birch lots of elongated small brooms	Broom tree leaf with gray spots	H	-	++++	ui	nt
**189**	Herttoniemi	HERS1 Large planted birch lots of elongated small brooms	Broom tree leaf with gray spots	H	-	++++	ui	nt
**190**	Herttoniemi	HERS2 Large planted birch less elongated small brooms	Broom tree leaf with chlorotic regions and gray spots	I	-	++++	ITC-A	nt
**191**	Herttoniemi	HERS2 Large planted birch less elongated small brooms	Broom tree leaf with chlorotic regions and gray spots	I	-	++++	ITC-A	nt
**192**	Herttoniemi	HERS2 Large planted birch less elongated small brooms	Broom tree leaf with chlorotic regions and gray spots	I	-	++++	ITC-A	nt
**193**	Herttoniemi	HERS2 Large planted birch less elongated small brooms	Broom tree leaf with chlorotic regions and gray spots	I	-	++++	ITC-A	nt

**194**	Herttoniemi	HERS2 Large planted birch less elongated small brooms	Broom tree leaf with chlorotic regions and gray spots	I	-	+++	ITC-A	nt
**195**	Herttoniemi	HERS2 Large planted birch less elongated small brooms	Broom tree leaf with chlorotic regions and gray spots	I	-	+++	ITC-A	nt
**196**	Herttoniemi	HERS2 Large planted birch less elongated small brooms	Broom tree leaf with chlorotic regions and gray spots	I	-	+++	ITC-A	nt
**197**	Herttoniemi	HERS2 Large planted birch, less elongated small brooms	Broom tree leaf with chlorotic regions and gray spots	I	-	+++	ITC-C	RGR-0
**198**	Herttoniemi	HERS2 Large planted birch, less elongated small brooms	Broom tree leaf with chlorotic regions and gray spots	I	-	++	ITC-D	RGR-3
**199**	Herttoniemi	HERS2 Large planted birch, less elongated small brooms	Broom tree leaf with chlorotic regions and gray spots	I	-	++	ITC-D	RGR-2
**200**	Herttoniemi	HERS2 Large planted birch, less elongated small brooms	Broom tree leaf with chlorotic regions and gray spots	I	-	+++	ITC-S	nt
**201**	Herttoniemi	HERS2 Large planted birch, less elongated small brooms	Broom tree leaf with chlorotic regions and gray spots	I	-	+++	ui	nt

**202**	Herttoniemi	HERS2 Large planted birch, less elongated small brooms	Broom tree leaf with chlorotic regions and gray spots	I	-	+++	ITC-F	nt
**203**	Herttoniemi	HERS2 Large planted birch, less elongated small brooms	Broom tree leaf with chlorotic regions and gray spots	I	-	+++	ITC-A	nt
**204**	Herttoniemi	HERS2 Large planted birch, less elongated small brooms	Broom tree leaf with chlorotic regions and gray spots	I	-	+++	ITC-A	nt
**205**	Herttoniemi	HERS2 Large planted birch, less elongated small brooms	Broom tree leaf with chlorotic regions and gray spots	I	-	+++	ITC-C	RGR-0
**206**	Herttoniemi	HERS2 Large planted birch, less elongated small brooms	Broom tree leaf with chlorotic regions and gray spots	I	-	+++	ITC-C	RGR-0
**207**	Herttoniemi	HERS2 Large planted birch, less elongated small brooms	Broom tree leaf with chlorotic regions and gray spots	I	-	++	ITC-C	RGR-0
**208**	Herttoniemi	HERS2 Large planted birch, less elongated small brooms	Broom tree leaf with chlorotic regions and gray spots	I	-	++	ui	nt
**209**	Herttoniemi	HERS2 Large planted birch, less elongated small brooms	Broom tree leaf with chlorotic regions and gray spots	I	-	+++	ITC-C	RGR-0

**210**	Herttoniemi	HERS2 Large planted birch, less elongated small brooms	Broom tree leaf with chlorotic regions and gray spots	I	-	+++	ITC-A	nt
**211**	Herttoniemi	HERS2 Large planted birch, less elongated small brooms	Broom tree leaf with chlorotic regions and gray spots	I	-	+++	ITC-D	RGR-2
**212**	Herttoniemi	HERS2 Large planted birch, less elongated small brooms	Broom tree leaf with chlorotic regions and gray spots	I	-	+++	ITC-C	RGR-0
**213**	Herttoniemi	HERS2 Large planted birch, less elongated small brooms	Broom tree leaf with chlorotic regions and gray spots	I	+++	+++	ITC-C	RGR-0
**214**		HERS2 Large planted birch, less elongated small brooms	Broom tree leaf with chlorotic regions and gray spots	I	-	+++	ITC-C	RGR-0
**215**	Herttoniemi	HERS2 Large planted birch, less elongated small brooms	Broom tree leaf with chlorotic regions and gray spots	I	-	+++	ITC-C	RGR-0
**216**	Herttoniemi	HERS2 Large planted birch, less elongated small brooms	Broom tree leaf with chlorotic regions and gray spots	I	+++	+++	np	nt
**217**	Herttoniemi	HERS2 Large planted birch, less elongated small brooms	Broom tree leaf with chlorotic regions and gray spots	I	-	+++	ITC-C	RGR-0
**218**	Vartioharju (VTH)	Large birch(hanging	Broom leaf gray spots and yellow	E	-	+++	ITC-D	RGR-0

		phenotype) with elongated brooms, heavily diseased(VTHS 1)	spot and deficient growth					
**219**	Vartioharju (VTH)	Large birch(hanging phenotype) with elongated brooms, heavily diseased(VTHS 1)	Broom leaf gray spots and yellow spot and deficient growth	E	-	+++	ITC-D	RGR-2
**220**	Vartioharju (VTH)	Large birch(hanging phenotype) with elongated brooms, heavily diseased(VTHS 1)	Broom leaf gray spots and yellow spot and deficient growth	E	-	+++	ITC-D	RGR-2
**221**	Vartioharju (VTH)	Large birch(hanging phenotype) with elongated brooms, heavily diseased(VTHS 1)	Broom leaf gray spots and yellow spot and deficient growth	E	-	++	ITC-F	nt
**222**	Vartiokylä	Large birch broom like tumors no shoot elongation from tumors (VTKS1).		G	-	+++	ui	nt

**223**	Herttoniemi	HERS1 Large planted birch lots of elongated small brooms near to gate	Surface sterilized leaf that was chopped	H’	+++	+++	ui	nt
**224**	Herttoniemi	HERS1 Large planted birch lots of elongated small brooms near to gate	Surface sterilized leaf that was chopped	H’	+++	+++	ui	nt
