# Supplementary material for: Distinct Taphrina strains from the phyllosphere of birch exhibiting a range of witches' broom disease symptoms
Source: Environ Microbiol. 2022 May 17;24(8):3549–64. doi: 10.1111/1462-2920.16037 (PMC9545635; doi:10.1111/1462-2920.16037)
Supplement: Supplementary file 8 — Table S3. Banding patterns observed with the RGR1 (Rco1 Gyp7 RsaI) CAPS (cleaved amplified polymorphic sequence) marker. [file EMI-24-3549-s006.pdf]

Supplemental Table S3. Banding patterns observed with the RGR1 (*Rco1 Gyp7* RsaI) CAPS (cleaved amplified polymorphic sequence) marker.

**RGR type**	**Banding pattern**
RGR-0	No PCR product
RGR-1	No digestion: 1 band: >1 kbp
RGR-2	Two bands: 650 bp, 550 bp
RGR-3	Three bands: 550 bp, 450 bp, 200 bp
