# Supplementary material for: Distinct Taphrina strains from the phyllosphere of birch exhibiting a range of witches' broom disease symptoms
Source: Environ Microbiol. 2022 May 17;24(8):3549–64. doi: 10.1111/1462-2920.16037 (PMC9545635; doi:10.1111/1462-2920.16037)
Supplement: Supplementary file 9 — Table S4. Cell size of 22 T. betulina selected strains. [file EMI-24-3549-s002.pdf]

Supplemental Table S4. Cell sizes of 22 *T. betulina* selected strains.

Type	Strains	Cell Size			
		Length (µm)	Significance group	Width (µm)	Significance groups
**I***	25	4.96±1.1	ab	3.39±0.8	abcde
	26	5.07±0.9	ab	3.5±0.7	ab
	31	4.75±1.3	ab	3.17±0.8	df
	34	4.9±0.9	ab	3.24 ±0.7	abce
	85	4.82±0.9	ab	3.51±0.7	abcdef
	112	5.12±0.9	ab	3.61±0.8	abce
	219	5.13 ±0.8	ab	3.5 ±0.7	abcdef
**II****	58	5.79±1.2	ac	3.58±0.8	acdef
	59	5.08±1.1	ab	3.7±0.9	abcdef
	62	4.95±1	ab	3.5±0.7	abcde
	63	4.81±0.9	ab	3.33±0.6	cdef
	68	4.96±0.87	b	3.45±0.7	b
	69	5.02±1.1	ab	3.39±0.6	abc
	82	5±1	d	3.41±0.7	cdef
	83	4.87±1	ab	3.27±0.7	df
	151	5.16±1.1	ab	3.4±0.7	abcde
	198	5.15±0.9	ab	3.69±0.8	abcde
	199	4.81±0.9	ab	3.3±0.6	abde
**III*****	11	5.65±1.2	cd	3.81±1.2	f
	19	4.88±1	ab	3.22±0.7	def
	20	5.28±1	ab	3.56±0.7	b
	129	5.08±1.1	ab	3.18±0.6	abcdef
